# Transcriptome Analysis Reveals Photoperiod-Associated Genes Expressed in Rice Anthers

**DOI:** 10.3389/fpls.2021.621561

**Published:** 2021-02-26

**Authors:** Shiyu Sun, Duoxiang Wang, Jingbin Li, Yaqi Lei, Gang Li, WenGuo Cai, Xiangxiang Zhao, Wanqi Liang, Dabing Zhang

**Affiliations:** ^1^Joint International Research Laboratory of Metabolic and Developmental Sciences, State Key Laboratory of Hybrid Rice, School of Life Sciences and Biotechnology, Shanghai Jiao Tong University, Shanghai, China; ^2^School of Agriculture, Food and Wine, University of Adelaide, Urrbrae, SA, Australia; ^3^Jiangsu Collaborative Innovation Center of Regional Modern Agriculture and Environmental Protection, Huaiyin Normal University, Huai’an, China

**Keywords:** transcriptome, rice anther, photoperiod, WGCNA, carbohydrate, transport, phytohormone, PGMS

## Abstract

Environmental conditions, such as photoperiod and temperature, can affect male fertility in plants. While this feature is heavily exploited in rice to generate male-sterile lines for hybrid breeding, the underlying molecular mechanisms remain largely unknown. In this study, we use a transcriptomics approach to identify key genes and regulatory networks affecting pollen maturation in rice anthers in response to different day lengths. A total of 11,726 differentially expressed genes (DEGs) were revealed, of which 177 were differentially expressed at six time points over a 24-h period. GO enrichment analysis revealed that genes at all time points were enriched in transport, carbohydrate, and lipid metabolic processes, and signaling pathways, particularly phytohormone signaling. In addition, co-expression network analysis revealed four modules strongly correlated with photoperiod. Within these four modules, 496 hub genes were identified with a high degree of connectivity to other photoperiod-sensitive DEGs, including two previously reported photoperiod- and temperature-sensitive genes affecting male fertility, *Carbon Starved Anthe*r and *UDP-glucose pyrophosphorylase*, respectively. This work provides a new understanding on photoperiod-sensitive pollen development in rice, and our gene expression data will provide a new, comprehensive resource to identify new environmentally sensitive genes regulating male fertility for use in crop improvement.

## Introduction

Hybrid breeding has made a great contribution to the yield increase of rice (*Oryza sativa*) relative to inbred varieties, with improvements of up to 20% (Khush, 2001; Michel et al., 2001; Yuan, 2004; Cheng et al., 2007). Originally, after the discovery of a wild abortive cytoplasmic male sterile (CMS) line in the 1970s, a three-line system containing the male-sterile line, a maintainer line, and a restorer line was used (Yuan, 1984). Due to the time-consuming and germplasm-limited nature of using separate maintainer and restorer lines (Chen et al., 2010), a more modern two-line system was developed in 1973, based on the discovery and application of environmentally sensitive genic male sterile lines (EGMS) (Shi, 1981). Photoperiod-sensitive (PGMS) lines and thermosensitive (TGMS) lines have been successfully developed and applied widely in rice breeding (Mou, 2016).

Since photoperiod is more stable and predictable than temperature, breeders have focused on PGMS lines. Several genes, such as *photoperiod-sensitive male sterility-1* (*pms1*) (Zhou et al., 2011; Fan et al., 2016), *pms2* (Zhang et al., 1994), *pms3* (Ding et al., 2012a,b; Zhou et al., 2012), *Programmed Cell Death 5* (*OsPDCD5*) (Wang Y. et al., 2010), and *Carbon Starved Anther* (*CSA*) (Zhang et al., 2013), have been reported to control PGMS. *Pms1* encodes a long non-coding RNA (lncRNA) that acts as a phased small-interfering RNA (phasiRNA)-generating locus to produce the *PMS1T* transcript (21-nt phasiRNA). *PMS1T* is highly expressed during pollen formation in wild-type plants under long-day (LD) conditions, and a mutation in *PMS1T* that causes an accumulation of phasiRNAs causes PGMS (Fan et al., 2016). A single-nucleotide polymorphism (SNP) in *pms3*, another lncRNA required for normal pollen development, reduces *pms3* transcript levels under LD conditions, leading to PGMS in a japonica cultivar, NK58S (Ding et al., 2012a,b). Subsequently, Zhou et al. (2012) identified the same SNP in NK58S-derived cultivars and reported the role of this mutation in producing one mutated small RNA, leading to PGMS and TGMS in *japonica* and *indica* backgrounds, respectively (Zhou et al., 2012). *OsPDCD5* is an ortholog of mammalian *programmed cell death 5*, whose decreased expression induces photoperiod-sensitive male sterility in rice under LD photoperiods (≥13.5 h sunlight) (Wang Y. et al., 2010). *CSA* encodes an R2R3 MYB transcription factor that regulates sugar accumulation in rice anther sink tissue; its knockout mutant accumulates insufficient sugar and starch levels in anthers under short-day (SD) conditions, resulting in male sterility (Zhang et al., 2010, 2013). A TGMS line currently used for hybrid seed production, *thermosensitive genic male sterile 5* (*tms5*), has been shown to contain a mutation in a conserved ribonuclease, RNase Z (Zhou et al., 2014). These findings reveal the complexity of the interaction between genetic components and environmental signals in determining male fertility in plants (Kim and Zhang, 2018). However, the global understanding of male reproduction in plants in response to different photoperiods remains largely unknown.

To comprehensively analyze molecular mechanisms controlling plant male fertility in response to photoperiod, we performed a global RNA sequencing (RNA-seq) analysis on anthers grown in LD and SD conditions. Genes associated with carbohydrate transport and metabolism, and phytohormone signaling, were highly correlated with day length during late pollen developmental. This work provides insights into the molecular mechanisms of photoperiod-dependent male fertility, which can lead to identification of new PGMS genes for hybrid breeding.

## Materials and Methods

### Plant Materials

Rice variety *O. sativa* L. *ssp. japonica* 9522 was grown in paddy fields around Shanghai (China) during the normal rice growing season (June–August). Photoperiod treatment was started after panicle initiation and continued until all samples were collected: LD was the natural daylight condition, with a photoperiod of ∼14 h; SD conditions were simulated by covering plants with black cloth for the last 2 h of natural daylight to create a 12-h photoperiod. Duplicate samples of anthers were collected at mitosis I (stage 11) based on anther length (about 2.2 mm), which was described by Zhang et al. (2011) at six time points (00:00, 04:00, 08:00, 12:00, 16:00, and 20:00; Supplementary Figure 1). All samples were frozen immediately in liquid nitrogen and stored at −80°C.

### RNA Sequencing

RNA isolation, library construction, and sequencing were performed by Novelbio Ltd (Shanghai, China). Briefly, TRIzol^®^ reagents (Invitrogen, Carlsbad, CA, United States) were used to isolate RNA from each of two biological samples from each experimental condition. RNA quality was assessed by 1% agarose gel electrophoresis, Nanodrop (ThermoFisher), and Bioanalyzer (Agilent 2100), with A260/A280 = 1.8–2.2, and RNA integrity number (RIN) > 6.5. Libraries were synthesized using the standard Ion proton RNA-Seq Kit v2.0, according to the manufacturer’s instructions. Library sequencing was conducted by Ion Proton^TM^ (Life Technologies, Carlsbad, CA, United States).

### Sequence Data Processing, Mapping, and Annotation

The genome and annotation of the rice reference cultivar Nipponbare was downloaded from the MSU Rice Genome Annotation Project^1^ (Kawahara et al., 2013). FastQC (Version 0.11.1) was used to remove reads with 20% of the base quality lower than 13. Clean reads were mapped to the reference genome using Mapsplice v2.1.8 and then read counts were calculated (Wang K. et al., 2010), which were normalized to reads per kilobase per million (RPKM) to obtain relative levels of transcript expression. Counts and RPKM for each gene are given in Supplementary Data Sheet 1. A summary of sequencing data for each sample is given in Supplementary Table 1 and Supplementary Figure 2. The sequencing data were also uploaded in NCBI (GSE163030). Expression data for genes of interest in other vegetative and reproductive tissues (Supplementary Figure 5B) were sourced from the Rice Expression Profile Database (RiceXPro) (Sato et al., 2012).

### Quantitative Reverse Transcription (qRT)-PCR Validation

cDNA was synthesized using the Fast RT Kit (Tiangen Biotech, Beijing), following the manufacturer’s instructions. qRT-PCR analysis was performed on the LightCycler 96 (Roche). The *OsActin* gene was used as internal control, and the relative expression level of target genes was calculated based on the 2^–ΔΔ*Ct*^ method (Livak and Schmittgen, 2001). Primer sequences for qRT-PCR were found in qPrimerDB (Supplementary Data Sheet 9) (Lu et al., 2017).

### DEG, GO, and KEGG Ontology Enrichment Analysis

Differentially expressed genes (DEGs) were identified by DEseq2, using | log_2_fold change| ≥ 1 and false discovery rate (FDR) < 0.05 (Audic and Claverie, 1997). The Venn diagram of all DEGs was plotted by Rpackage “venn” (Adrian, 2019), while heatmaps and dendrograms of gene expression were plotted with Rpackage “pheatmap” (Raivo Kolde, 2019). The cluster of all DEGs was also visualized by “pheatmap” based on k-means clustering algorithm. Gene ontology (GO) enrichment was performed by agriGo^2^, and significant enriched GO terms were defined with *p* value < 0.01 and *q* value < 0.05 (Tian et al., 2017). The visualization of GO enrichment results was performed by Rpackage “Clusterprofiler” (Yu et al., 2012). KEGG (Kyoto Encyclopedia of Genes and Genomes) enrichment was also performed using Rpackage “Clusterprofiler” (Kanehisa and Goto, 2000).

### Construction of Gene Co-Expression Networks

Weighted gene co-expression network analysis R package (version 3.6) (Langfelder and Horvath, 2008) was used to construct co-expression networks of all expressed genes with automatic, one-step network construction and module detection. First, the soft thresholding power β was chosen based on the lowest power for which the scale-free topology fit index reached a high value. The function “blockwiseModules” was then used to construct topological overlap matrix (TOM) and module detection. The associations between modules and trait were estimated using Pearson correlation coefficient between module eigengenes and phenotype where 0/1 were used to define SD/LD or light/dark conditions. The expression patterns of four trait-related modules were plotted by “ggplot2” (Wickham, 2016). Hub genes were identified by both gene significance and module membership. The module membership (MM) is the correlation of gene expression profile with module eigengenes, and gene significance (GS) is the association of individual genes with that trait. Hub genes were set to a threshold of GS and MM > 0.8 (Horvath and Dong, 2008).

### Visualization of Co-expression Genes

Cytoscape (V3.7.2) (Shannon et al., 2003) was used to visualize co-expression networks. Input data from turquoise module were chosen based on the weight in terms of correlation value from the TOM (Topological Overlap Matrix Value), so that a higher value refers to a strong co-expression. The top 100 highest correlations with *CSA* and *Ugp1* genes were chosen and highlighted the top 1% correlation value of genes.

## Results

### DEGs in SD and LD Anthers

A total of 11,726 unique DEGs were identified between SD and LD conditions (Supplementary Data Sheet 2). Six genes from the RNA-seq data were selected for confirmation by quantitative reverse-transcription PCR (qRT-PCR), and patterns of expression with the two methods were found to be consistent (Supplementary Figure 3).

**FIGURE 1:**
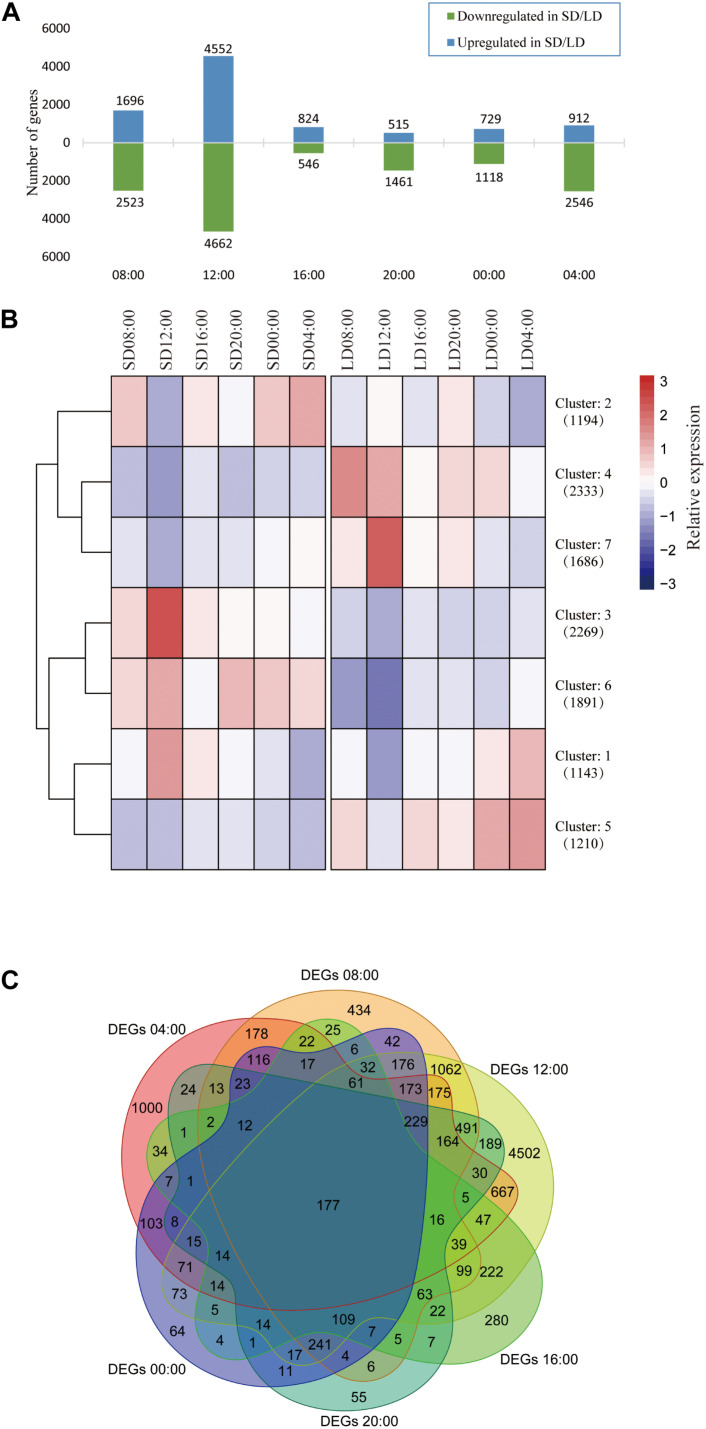
Expression of 11,726 DEGs in response to photoperiod conditions. SD, short day; LD, long day. **(A)** Number of up- and down-regulated DEGs at each time point. **(B)** Heatmap and dendrogram of all DEGs in all samples (*k* = 7). Gene numbers in each cluster given in brackets. DEGs assembled to seven clusters: three clusters up-regulated in SD (2, 3, and 6); three clusters up-regulated in LD (4, 5, and 7); and Cluster 1, with higher daytime expression under SD conditions, and nighttime expression under LD conditions. The value of center point of seven clusters represents relative expression. **(C)** Venn diagram showing all DEGs at six time points. A total of 177 genes showed differential expression between SD and LD at all time points.

The highest numbers of DEGs were expressed at 12:00, with 4552 genes up-regulated and 4662 genes down-regulated in SD anthers compared with LD anthers (Figure 1A and Supplementary Figure 4). Interestingly, the lowest number of DEGs was observed 4 h later at 16:00, with only 824 up-regulated and 546 down-regulated genes in SD anthers. All 11,726 DEGs assembled into just seven clusters, confirming that the largest differences in expression between LD and SD appeared at 12:00, followed by 08:00 and 04:00 (Figure 1B). Additionally, the 12:00 time point saw expression of the largest number of unique DEGs (4502), whereas the least numbers of unique DEGs occurred at 20:00 (55) and 00:00 (64) (Figure 1C). These results suggest that photoperiod dramatically affects gene expression patterns in anthers, with the largest changes occurring in the morning (04:00, 08:00, and 12:00), even before dawn.

#### Anther DEGs at All Time Points

A total of 177 genes were differentially expressed at all time points in SD and LD anthers (Figure 1C). Most of these genes (138) were up-regulated in LD anthers, with low or undetectable expression in SD anthers (Supplementary Figure 5A). A large proportion of these DEGs (51 genes, 29%) exhibit significantly higher expression in the anther than in other rice tissues (Supplementary Figure 5B), including previously reported anther-related genes such as *ATP Binding Cassette G15* (*OsABCG15*) (Wu et al., 2014), *LESS ADHESIVE POLLEN 6* (*OsLAP6*) (Zou et al., 2017), and *HOTHEAD-Like1* (*HTH1*) (Liu et al., 2016). These 177 DEGs were found in GO categories primarily associated with cell wall synthesis and metabolism, including hydrolytic enzyme expression and regulation, indicating that the anther cell wall structure and composition may be photoperiod-sensitive (Supplementary Figure 5C). These genes may play key roles in anther development and pollen formation, and can be used as markers to distinguish SD and LD conditions.

### Functional Analysis of DEGs in SD and LD Anthers

**FIGURE 2:**
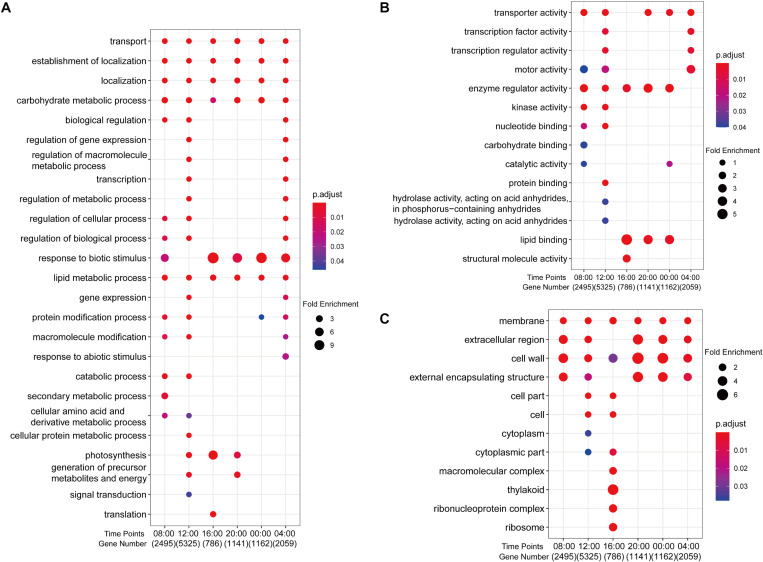
Enriched GO terms of 11,726 DEGs between SD and LD rice anther samples. **(A)** Biological processes. **(B)** Molecular function. **(C)** Cell component. Fold enrichment, depicted by circle size, which calculated by GeneRatio/BgRatio; p.adjust, shown by color, indicates significance level of enrichment results from blue to red.

Gene ontology and kyoto encyclopedia of genes and genomes functional analysis of anther DEGs indicated that genes involved in transport, lipid and carbohydrate metabolism, and metabolic regulation were most affected by photoperiod (Figure 2 and Supplementary Figure 6). GO enrichment analysis revealed that DEGs at all time points were enriched in GO “biological process” categories of “transport,” “localization,” “establishment of localization” (transport is a sub-item of these two terms), “carbohydrate metabolic process,” and “lipid metabolic process” (Figure 2A and Supplementary Data Sheet 3). DEGs involved in regulatory processes were generally enriched at 12:00 and 04:00, while metabolic processes were enriched at 08:00 and 12:00 (Figure 2A). In “molecular function” categories, the GO terms of “transport activity” and “enzyme regular activity” were enriched at most time points except 16:00 and 04:00, respectively, while “catalytic activity” was enriched at 08:00 and 00:00 (Figure 2B). For the “cell component” GO category, DEGs associated with “membrane” and “cell wall” were seen at all time points, while specific cellular components were most affected at 12:00 and 16:00 (Figure 2C).

KEGG analysis showed that genes encoding proteins involved in “pentose and glucuronate interconversions” in carbohydrate metabolism were enriched at 08:00, 12:00, 20:00, and 00:00 (Supplementary Data Sheet 4 and Supplementary Figure 6). *UDP-glucose pyrophosphorylase 2* (*OsUgp2*), a reported TGMS gene required for starch accumulation in pollen (Mu et al., 2009), was present in this pathway. In addition, genes associated with “starch and sucrose metabolism” and “carbon metabolism” were specifically enriched at 12:00, indicating a major difference in carbohydrate metabolism between SD and LD anthers at this time. Genes encoding “photosynthesis-antenna proteins” were enriched at all time points except 08:00. Rice *chlorophyll a/b* binding protein gene, *OsCAB1R*, a circadian clock-controlled gene (Sugiyama et al., 2001), is part of this pathway, indicating that rhythm genes may play photoperiod-sensitive roles in the anther. Calmodulin-related genes were mainly enriched in “plant–pathogen interaction” at 08:00, 20:00, and 04:00, such as *calcium-dependent protein kinase 9* (*OsCPK9*), *OsCPK21*, and *OsCPK25/26*. *OsCPK9* overexpression has been shown to improve pollen fertility under drought stress and shows sensitivity to ABA (Wei et al., 2014). Specific enrichment of mitogen-activated protein kinase (MAPK) pathway genes involved in defense against biotic stress was observed at 04:00.

#### DEGs Involved in Transport and Carbohydrate and Lipid Metabolism

**FIGURE 3:**
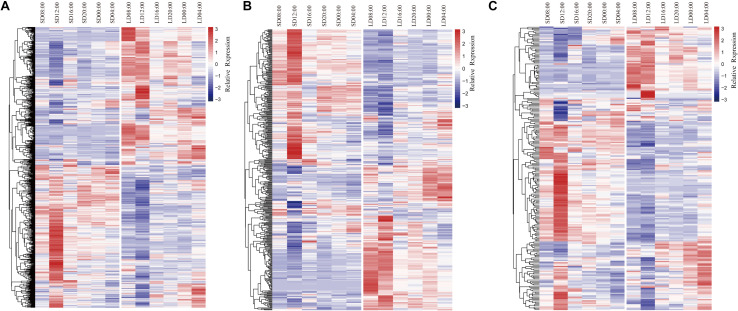
Heatmaps and dendrograms of DEGs encoding proteins involved in **(A)** transport; **(B)** carbohydrate metabolism; **(C)** lipid metabolism. Blue color indicates down-regulation in anthers; red color indicates up-regulation in anthers.

Genes involved in transport were differentially expressed in anthers at all time points under the two photoperiod conditions, including 189 genes encoding proteins involved in ion transport, 82 genes associated with protein transport, and 54 genes associated with lipid transport (Figure 3A and Supplementary Data Sheet 5). These genes could be grouped in two main clusters that were generally up-regulated in either LD or SD conditions. Genes up-regulated in SD anthers were generally most highly expressed at 12:00, but genes up-regulated in LD anthers had high expression at the 08:00 time point as well. For ion transport, DEGs predicted to encode potassium transporters, including high-affinity K^+^ transporter (HKT) (Jabnoune et al., 2009) and HAK family (Bañuelos et al., 2002) proteins, as well as sodium, calcium, hydrogen (proton), iron, boron, and zinc transporters were represented at all time points. For protein transport, genes encoding 15 rat sarcoma-related transporter and 17 mitochondrial translocase proteins were differentially expressed. A small GTPase protein, OsRacD, which has been shown to participate in light signal transduction and the fertility switch of NK58S (Ye et al., 2004), was more highly expressed under SD conditions at 08:00 and 12:00. For lipid transport, DEGs encoding 44 lipid transfer protein (LTP) family members were detected. Among these was *Photoperiod-sensitive dwarf 1* (*Psd1*), a non-specific *LTP* gene whose mutation can cause dwarfism under LD and low-temperature conditions (Deng W. et al., 2020). *Psd1* was up-regulated under SD conditions at 12:00. Other transport-associated DEGs included sugar transporters, such as monosaccharide transporters (MSTs) and sucrose transporters (SUTs). For example, *MST7* was up-regulated under SD conditions at all time points, while *MST5* and *SUT1* were up-regulated under LD conditions at all times except 16:00.

In the GO category “carbohydrate metabolism,” DEGs encoding glycosyl hydrolase and transferase proteins were highly represented, including 250 hydrolases and 118 transferases (Figure 3B and Supplementary Data Sheet 5). These genes again clustered into two groups, each up-regulated under one of the two photoperiod conditions, and genes up-regulated under SD conditions were again most highly expressed at 12:00 (Figure 3B). DEGs up-regulated under LD conditions exhibited two patterns of expression: a larger group most highly expressed at 08:00 and 12:00 pm, while a second, smaller group exhibited peak expression at 00:00 and 04:00 (Figure 3B). *Hexokinase* (*HXK*) family genes including *HXK5* (Cho et al., 2009), *HXK7* (Kim et al., 2016), and *HXK10* (Xu et al., 2008) as well as four invertases (*INV*) (Oliver et al., 2005; Deng X. et al., 2020) were identified, and these were spread across all three major patterns of expression.

DEGs encoding proteins involved in lipid metabolism related genes also exhibited three distinct patterns of expression (Figure 3C and Supplementary Data Sheet 5). Genes generally up-regulated in SD anthers had maximum expression at 12:00; genes up-regulated in LD anthers generally peaked at 08:00 and 12:00, while a third group of genes exhibited maximum expression in SD anthers at 12:00 and in LD anthers at 04:00. Two genes associated with male sterility, *Wax-deficient anther 1* (*Wda1*) and *HUMIDITY-SENSITIVE GENIC MALE STERILITY 1* (*HMS1*) (Jung et al., 2006; Chen et al., 2020), were among the genes up-regulated in SD anthers. *Wda1* is associated with biosynthesis of very-long-chain fatty acids for the formation of anther epicuticular wax crystals, whose mutation causes complete male sterility (Jung et al., 2006). *HMS1* encodes a β-ketoacyl-CoA synthase, and its mutant is humidity-sensitive genic male sterile (Chen et al., 2020).

#### DEGs Involved in Circadian Rhythm

Based on our finding that *OsCAB1R*, a circadian clock-controlled gene, was a DEG in the “photosynthesis-antenna proteins” GO class (Supplementary Data Sheet 4), we analyzed, and found, differential expression of other genes known to be affected by circadian rhythm (Supplementary Figure 7 and Supplementary Data Sheet 5). Rice *CIRCADIAN CLOCK ASSOCIATED 1* (*OsCCA1*), homologous to Arabidopsis *AtCCA1* (Izawa et al., 2002), showed a higher expression in the early morning, with almost twofold higher expression under LD compared with SD conditions at 04:00; *ZEITLUPE 2* (*ZTL2*) had a similar pattern but peaked under LD at 08:00. At 12:00, *OsCCA1* was expressed nearly seven times higher in SD compared with LD anthers. Rice *PSEUDO-RESPONSE REGULATOR* (*OsPRR*), *OsPRR95*, homologous to Arabidopsis circadian oscillator *AtPRR5* and *AtPRR9* (Murakami et al., 2005), showed fivefold expression higher at 12:00 under SD compared with that of LD conditions. *OsPRR37* shows up-regulation at night under LD (20:00, 00:00, and 04:00), four times higher at 00:00 and 04:00, while *OsPRR73*, Early flowering 3 (*OsELF3*), and *ZTL1* showed little difference between SD and LD conditions. Rice *LUX ARRHYTHMO* (*OsLUX*), homologous to a nighttime repressor of circadian gene expression *AtLUX* (Murakami et al., 2007), was also up-regulated at 16:00 in SD conditions. Other rice circadian clock or flowering genes *FLAVIN BINDING, KELCH REPEAT, F-BOX1* (*OsFKF1*), *TIMING OF CAB EXPRESSION 1* (*OsTOC1*), and *Gigantea* (*OsGI*) (Hayama et al., 2003; Murakami et al., 2003) were expressed more highly at 16:00 in LD anthers than in SD anthers, although by less than a twofold difference. These results indicated that rhythm genes in the anther are sensitive to photoperiod, particularly under SD conditions.

### Construction of Co-expression Gene Networks

To examine the regulatory networks of genes under SD and LD conditions, 39,966 genes (99.8%) were categorized into 34 modules based on scale-free topology model (β = 7; Supplementary Figure 8). The remaining 78 genes that did not assemble into existing modules were grouped into a 35th “module” (gray). Standard modules ranged in size from 177 to 5852 genes, e.g., black contained 1496 genes that generally expressed highly at LD 12:00; dark red had 555 genes that generally expressed highly at SD 16:00 (Supplementary Table 2 and Supplementary Figure 9B).

#### Identification of LD- and SD-Related Modules

**FIGURE 4:**
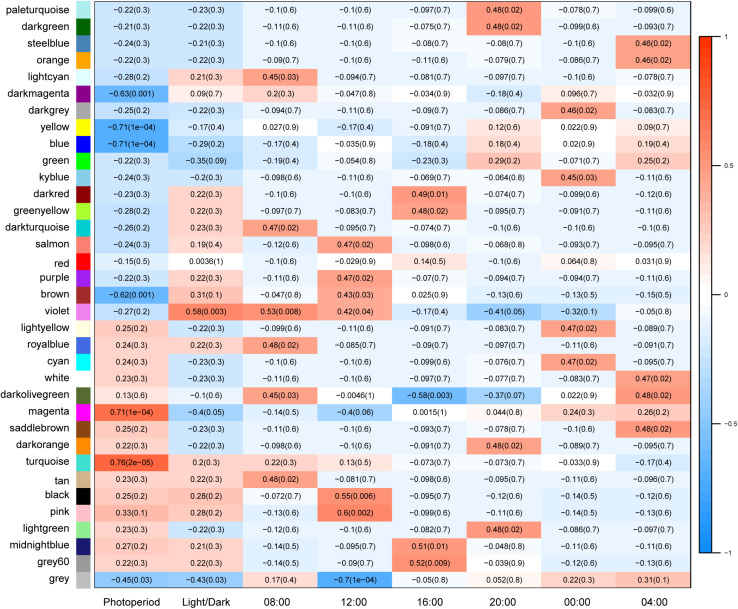
Module-trait associations. Correlation coefficients between different modules and traits show in matrix. Each cell contains correlation coefficient with *p* value in bracket. Photoperiod, short-day, and long-day condition; LD acts as positive control, in which red color shows positive correlation with LD; SD is the opposite; Light and Dark: daytime vs. night conditions, daytime is defined as positive blank including samples at 08:00, 12:00, and 16:00; night includes 20:00, 00:00, and 04:00.

Variation in gene expression was higher between SD and LD conditions than between dark/light conditions (Figure 4), suggesting that photoperiod has a significant effect on anther development. Expression of 36% of genes correlated with SD or LD conditions, suggesting that a large proportion of genes responded directly or indirectly to day length. Gene expression in four modules in particular was observed to correlate significantly (*p* < 0.05) with photoperiod: genes in turquoise (5852 genes, *r* = 0.76) and magenta (1132 genes, *r* = 0.71) modules were up-regulated in LD, while genes in yellow (4929 genes, *r* = −0.71), and blue (2,698 genes, *r* = −0.71) modules were up-regulated in SD (Supplementary Table 2, Supplementary Figure 9, and Supplementary Data Sheet 6).

#### Functional Specific Enrichment Analysis of Related Modules

**FIGURE 5:**
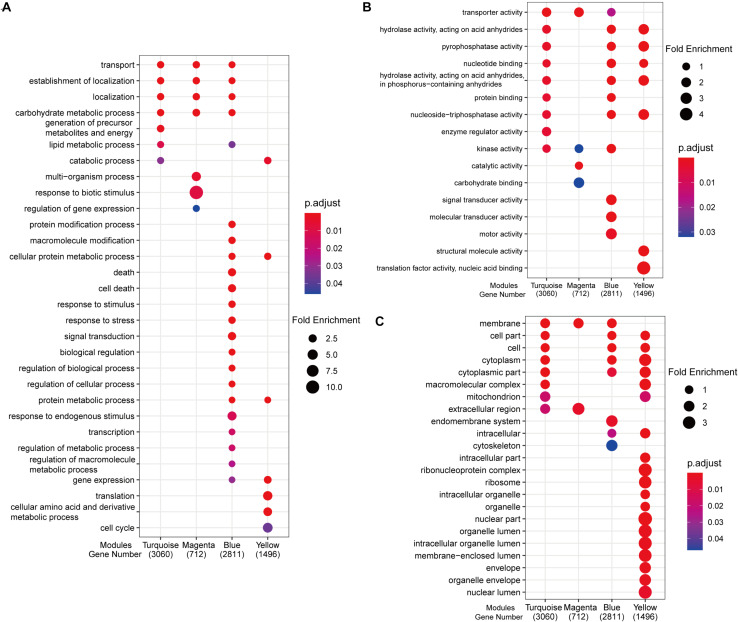
Enriched GO terms of genes in four co-expression modules. **(A)** Biological processes. **(B)** Molecular function. **(C)** Cell component. Fold enrichment, depicted by circle size, which is calculated by GeneRatio/BgRatio; p.adjust, shown by color, indicates significance level of enrichment results from blue to red.

Gene ontology analysis revealed enrichment of different functional categories of genes among modules (Figure 5). The turquoise, magenta, and blue modules were enriched for biological processes including “transport,” “localization,” “establishment of localization,” and “carbohydrate metabolic process,” and contained genes encoding various potassium (HKT and HAK family) and sugar (MST, SUT) transporters, and carbohydrate metabolic proteins such as sucrose synthase (SUS) and invertases (Supplementary Data Sheet 7).

Genes associated with “lipid metabolic process” were enriched in turquoise and blue modules (Figure 5A), which is also consistent with the GO enrichment of DEGs (Figure 2A). The blue module was enriched in more GO categories than other modules, including “protein metabolic process” and “macromolecule modification” (Figure 5A). In the “protein metabolic process” category were TGMS genes, such as *Temperature-sensitive Male Sterile 10* (*TMS10*) and *TMS10-Like gene* (*TMS10L*) (Yu et al., 2017) and *ubiquitin fusion ribosomal protein L40* (*UbL404*), which acts downstream of *TMS5* (Zhou et al., 2014), as well as genes encoding light-responsive proteins, such as photoreceptors *Phytochrome B(PHYB) and PHYC*, and *Heading date 6* (*Hd6*) (Takahashi et al., 2001; Takano et al., 2005) (Supplementary Data Sheet 7).

KEGG enrichment analysis revealed genes associated with “oxidative phosphorylation,” the “TCA (tricarboxylic acid) cycle,” and “carbon metabolism pathway” in the turquoise module (Supplementary Figure 10). The magenta module was enriched in diterpenoid biosynthesis, while yellow module was enriched in categories associated with DNA replication, RNA transport, and ribosome biosynthesis. These results indicate that protein metabolism and transcription/translation processes are also affected by day length.

#### Identification of Hub Genes

Hub genes are those with a high degree of connectivity and co-relationships with genes in modules of interest for a given phenotype (Langfelder and Horvath, 2008). Based on the module membership and gene significance, 239, 62, 139, and 56 hub genes were identified in turquoise, magenta, blue, and yellow modules, respectively (Supplementary Data Sheet 6). As identified in our earlier GO and KEGG analyses, these genes were involved a broad range of processes, including macromolecule, carbohydrate, and lipid metabolisim and transport. Of particular interest, six hub genes have previously been reported to be involved in male sterlity or low fertility, including LOC_Os01g16810 (*CSA*) (Zhang et al., 2010, 2013), LOC_Os09g38030 (*UDP-glucose pyrophosphorylase gene 1*, *OsUgp1*) (Chen et al., 2007), LOC_Os01g54620 (*cellulose synthase catalytic subunit genes 4, OsCesA4*) (Tanaka et al., 2003), LOC_Os02g04840 (*collapsed abnormal pollen 1*, *CAP1*) (Ueda et al., 2013), LOC_Os04g39980 (*dioxygenase for auxin oxidation gene*, *DAO*) (Zhao et al., 2013), and LOC_Os06g04090 (*secondary wall NAC 1*, *OsSWN1*) (Chai et al., 2015). The discovery of known key regulators of pollen sterility indicates the robustness of our co-expression and hub gene construction method to mine for key anther regulator genes. *CSA* encodes a key R2R3 MYB transcription factor that controls male fertility in a photoperiod-dependent manner (Zhang et al., 2013). *OsUgp1* is required for callose depostion in pollen mother cells in meiosis; knockdown of *OsUgp1* causes a thermosensitive male sterile defect (Chen et al., 2007). *OsCesA4* is required for cellulose synthesis, and its mutant shows brittle culm and low fertility (Tanaka et al., 2003). *CAP1* encodes an arabinokinase-like protein required for nucleus, cytoplasm, and intine cell wall development in rice pollen. *DAO* encodes a 2-oxoglutarate-dependent-Fe (II) dioxygenase essential for anther formation and dehiscence, pollen fertility, and seed initiation (Zhao et al., 2013). *OsSWN1* is a NAC transcription factor gene involved in secondary cell wall biosynthesis, whose down-regulation causes a decrease in lignin and an increase in polysaccharide content in cell walls, resulting in anther dehiscence defects (Chai et al., 2015). The identification of these genes in our analyses suggests that these previously characterized pollen development regulators could also have a day length-dependent role.

An additional 30 hub genes encoding transcription factors were detected, including 5 previously unreported MYB proteins and 10 zinc-finger proteins (Table 1). The function of these unreported genes in regulating anther development needs to be further characterized, but it seems likely that they will be key regulators of rice anther development particularly affected by photoperiod.

**TABLE 1:** Transcription factor hub genes in four modules.

**Gene ID**	**Module**	**Annotation**	**Arabidopsis ortholog**
LOC_Os01g50720	Blue	MYB family transcription factor, putative, and expressed	MYB86
LOC_Os02g35144	Blue	Zinc finger, C3HC4-type domain-containing protein, and expressed	AT4G10150
LOC_Os03g11614	Blue	OsMADS1	AT3G02310
LOC_Os03g13600	Blue	ZOS3-07 – C2H2 zinc finger protein, expressed	ZFP4
LOC_Os03g51910	Blue	Basic helix-loop-helix protein, putative, and expressed	AT1G10120
LOC_Os03g53050	Blue	WRKY121, expressed	WRKY39
LOC_Os08g26880	Blue	bZIP transcription factor domain-containing protein, expressed	bZIP44
LOC_Os09g36730	Blue	OsMYB108	MYB4
LOC_Os12g07120	Blue	GATA zinc finger domain-containing protein, expressed	GATA14
LOC_Os12g32620	Blue	OsWLIM1 – LIM domain protein, putative actin-binding protein and transcription factor, and expressed	WLIM1
LOC_Os02g33560	Magenta	Expressed protein	AT5G04840
LOC_Os03g21060	Magenta	Plant-specific NAC transcriptional activator, OsNAP	NAC047
LOC_Os06g09310	Magenta	Zinc finger, C3HC4-type domain-containing protein, and expressed	ATL3
LOC_Os09g32730	Magenta	Zinc finger, C3HC4-type domain-containing protein, and expressed	AT3G25030
LOC_Os10g33760	Magenta	No apical meristem protein, putative, and expressed	NAC074
LOC_Os01g16810	Turquoise	CSA	MYB105
LOC_Os01g16950	Turquoise	Zinc finger, C3HC4-type domain-containing protein, and expressed	AT1G55530
LOC_Os01g51260	Turquoise	MYB family transcription factor, putative, and expressed	MYB26
LOC_Os01g68900	Turquoise	Zinc finger, C3HC4-type family protein, and expressed	AT4G13100
LOC_Os02g44120	Turquoise	ZOS2-13 – C2H2 zinc finger protein, and expressed	AT1G02040
LOC_Os04g31804	Turquoise	OsMADS64 – MADS-box family gene with M-alpha type-box, expressed	AGL79
LOC_Os04g56990	Turquoise	REGULATOR OF LEAF INCLINATION1	AT5G06800
LOC_Os06g04090	Turquoise	Secondary wall NAC 1, OsSWN1	EMB2301
LOC_Os07g02800	Turquoise	MYB family transcription factor, putative, and expressed	AT2G03500
LOC_Os09g26420	Turquoise	AP2 domain-containing protein, expressed	RAP2.12
LOC_Os12g04590	Turquoise	Zinc finger, C3HC4-type domain-containing protein, and expressed	AT3G19950
LOC_Os12g33070	Turquoise	OsMYB46	MYB46
LOC_Os06g40330	Yellow	MYB family transcription factor, putative, and expressed	MYB101
LOC_Os11g03540	Yellow	AP2 domain-containing protein, expressed	WRI1
LOC_Os12g42970	Yellow	GATA zinc finger domain-containing protein, expressed	GATA2

#### Genes Highly Co-expressed With *CSA* and *Ugp1*

**FIGURE 6:**
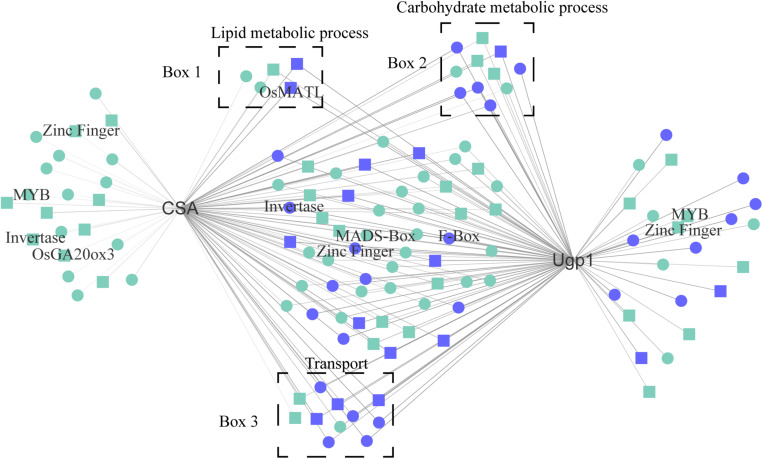
High co-expressed genes of *CSA* and *Ugp1.* Blue indicates the top 1% TOM value genes; rectangle represents hub genes. *OsGA20ox3* (*gibberellin 20 oxidase 3*, LOC_Os07g07420); *OsMATL* (*MATRILINEAL*, LOC_Os03g27610); *CSA* (*Carbon Starved Anther*, LOC_Os01g16810); *Ugp1* (*UDP-glucose pyrophosphorylase*, LOC_Os09g38030); *Invertase* (LOC_Os12g37480, LOC_Os02g01310); MYB-TFs (LOC_Os01g51260, LOC_Os06g46560); MADS-box genes (LOC_Os06g11970); Zinc Finger genes (LOC_Os02g44120, LOC_Os01g68900, and LOC_Os12g08070); F-box (LOC_Os04g19800); Box 1, lipid metabolic process-related genes; Box 2, carbohydrate metabolic process-related genes; Box 3, Transport-related genes.

Two hub genes, *CSA* and *Ugp1*, have been reported to be involved in rice photoperiod-sensitive and thermosensitive male fertility, respectively. We visualized genes with high correlation to *CSA* and *Ugp1* (Figure 6 and Supplementary Data Sheet 8). Notably, *CSA* and *Ugp1* shared 74 overlapped correlation genes, such as *MATRILINEAL* (*OsMATL*) and *OsHXK5* (Cho et al., 2009; Yao et al., 2018), which were up-regulated in LD anthers, unlike that of *CSA* and *Ugp1*—as undirected network relationships. Four co-expressed genes were also selected for qRT-PCR analysis and confirmed this prediction outcome (Supplementary Figure 11). *OsMATL* encodes a pollen-specific phospholipase that is involved in haploid induction, whose knockout mutant displays reduced seed set (Yao et al., 2018). *OsHXK5* encodes a hexokinase that functions as a glucose sensor. Plants overexpressing *OsHXK5* showed a hypersensitive and slow growth phenotype (Cho et al., 2009). Six TFs such as MYB, Zinc Finger, and MADS-Box proteins were in network; 5 lipid metabolic-associated genes, 11 carbohydrate metabolic genes, and 11 transport genes were also detected. In addition, there were 38 hub genes highly co-expressing with *CSA* and *Ugp1*. These results indicate that *CSA* and *Ugp1* are likely to share regulatory pathways in controlling environmentally mediated male development in rice.

## Discussion

Transcript profiling of anthers grown under different photoperiod conditions provides information to help understand the molecular mechanisms that affect male fertility in response to day length and thus improve our ability to create new PGMS lines for rice breeding. In this study, we have conducted the first genome-wide transcriptomic and gene network analysis of anthers grown under short and long day lengths and revealed functional and regulatory genes that respond to photoperiod.

### Photoperiod Changes Gene Expression in Anthers

Approximately one-third of expressed genes (11,726 out of ∼30,000 in different samples) were differentially expressed in anthers grown under SD and LD conditions, with gene expression generally down-regulated under SD conditions (Figure 1A, Supplementary Table 1, and Supplementary Data Sheet 2). A total of 177 genes were differentially expressed at all time points (Figure 1C), of which 51 were specifically expressed in the anther and other reproductive tissues (Supplementary Figure 5B and Supplementary Data Sheet 2). Several genes whose mutation is known to cause male sterility were found in this set of 51 genes. *OsABCG15* plays a role in the formation of the anther cuticle and pollen exine and is involved in the export of lipid precursors from the tapetum to anther locules (Wu et al., 2014; Zhao et al., 2015). *OsLAP6* functions in sporopollenin metabolism and affects bacula elongation to regulate pollen exine formation (Zou et al., 2017). *HOTHEAD 1* (*HTH1*) encodes a glucose–methanol–choline (GMC) oxidoreductase; its RNAi line and mutant both display defective anther walls and aborted pollen with decreased amounts of long-chain fatty acids and cutin (Xu et al., 2017). Other genes in this set of 177 DEGs, especially genes that specifically expressed in anthers, may also affect male fertility, particularly in a photoperiod-sensitive manner.

#### DEGs Associated With Transport and Metabolism

Gene ontology enrichment analysis of DEGs at different time points revealed a high proportion of genes associated with transport and metabolism, particularly of ions, carbohydrates, and lipids (Figure 2). Generally, these genes were up-regulated at 12:00 under SD and at 08:00 and 12:00 under LD conditions, indicating that these pathways may be associated with the growth and physiological adaption of plants in response to photoperiod.

In stage 11 anthers, mitosis and accumulation of nutrients are the main developmental processes, consistent with our GO enrichment results. Correct ion homeostasis is essential for normal plant growth; accordingly, many genes encoding ion transporters were detected (Supplementary Data Sheet 3). Several potassium transporter family genes were enriched, like *HKT*, *HAK*, and *AKT* family genes (Rodríguez-Navarro and Rubio, 2006). *HKT1;4* can also exclude Na^+^ in productive stage of rice (Suzuki et al., 2016). *Cation/H^+^ exchanger 14* (*OsCHX14*), involved in K^+^ homeostasis, may play an important role during rice flowering stages (Chen et al., 2016). *OsBOR1* and *OsBOR4* DEGs encode boron transporters; pollen from *osbor4* mutants have defects in pollen tube elongation (Nakagawa et al., 2007; Tanaka et al., 2013). *OsFRDL1* is a MATE (multidrug and toxic compound extrusion) family gene, a citrate transporter of iron, whose mutant exhibits a decrease in pollen viability and grain fertility (Yokosho et al., 2009, 2016). *SULTR-like phosphorus distribution transporter* (*SPDT*) encodes a plasma-membrane-localized phosphorus transporter that preferentially allocates phosphorus to grains (Yamaji et al., 2017). *Psd1* encodes a non-specific lipid transfer protein (nsLTP) that regulates cell division and elongation; *psd1* mutants show photoperiod-thermosensitive dwarfism (Deng W. et al., 2020).

Many DEGs were also found to encode sugar transporters and catabolic proteins, such as monosaccharide transporters (*MST1* to *MST8*), sucrose transporters (*SUT1*, *SUT2*, *SUT3*, and *SUT5*), and invertases (*INV1*, *INV2, INV3*, and *INV4*). Proteins from these three family genes cover sucrose transport from source to sink. Sucrose is loaded into the phloem by SUTs and unloaded in sink tissues, like anthers or seeds, where sucrose is converted by invertases into hexoses and imported into cells by MSTs (Lim et al., 2006). OsMST4 has been shown to play a role in monosaccharide supply during grain filling (Wang et al., 2007), and *OsMST5* is expressed in panicles before pollination and may be involved in pollen development (Ngampanya et al., 2003). MST8 is predicted to transport monosaccharides from anther wall layers to the microspore, affecting the unloading process of the source–sink tissue during male reproduction (Zhang et al., 2010). SUT1 is predicted to play a role in the uptake of sucrose from the apoplast, and its lack during anther development suppresses pollen germination (Hirose et al., 2010). *INV4* is expressed specifically in the anther, down-regulated by cold stress, resulting in abnormal accumulation of sucrose (Oliver et al., 2005).

Other DEGs associated with carbohydrate metabolism could also affect anther viability. *Glucan synthase-like 5* (*GSL5*) encodes a callose synthase, and its mutant showed decreased fertility due to defects in the pollen callose wall (Shi et al., 2015). *Oryza sativa glucanase 1* (*Osg1*) encodes a plant β-1,3-glucanase essential for callose degradation during tetrad dissolution; RNAi plants exhibited delayed release of young microspores into anther locules, causing male sterility (Wan et al., 2011). Hexokinase (HXK) family proteins are involved in sugar sensing and signaling (Cho et al., 2006), and genes encoding HXK5, HXK7, and HXK10 were among DEGs detected in our analysis. HXK10 can also phosphorylate hexose sugars; RNAi knockdown lines showed non-dehiscent anthers (Xu et al., 2008). Genes encoding disproportionating enzymes *DPE1* and *DPE2* were also among our DEGs; these proteins are involved in starch metabolism and maturation of starch granules (Akdogan et al., 2011; Hwang et al., 2016).

Differentially expressed genes encoding transport and metabolic genes clustered into two, similarly sized groups that were up-regulated in either SD or LD anthers, suggesting that these biological processes may occur, or be regulated by, different pathways depending on photoperiod to maintain nutrient balances essential for normal development (Figures 3A–C). While previously reported genes affecting male sterility were found among our photoperiod-sensitive DEGs, their mutations generally caused total male sterility rather than PGMS (i.e., *Osg1*, *OsFRDL1*, and *OsBOR4*). Discovery of new potential PGMS regulators will require careful analysis of differential expression of candidate genes under SD and LD conditions.

#### Differential Expression of Circadian Genes

We analyzed the expression pattern of genes associated with the control of circadian rhythm and found that *OsLUX*, *OsFKF1*, *OsGI*, and *OsTOC1* show higher expression in LD over SD anthers at16:00, *OsPRR37* was up-regulated under night at LD, while *OsPRR95* has higher expression at 12:00. *OsCCA1* was up-regulated at 12:00 and 16:00 in SD compared with LD anthers (Supplementary Figure 7). These genes and proteins interact with each other in complex ways. In *Arabidopsis*, *CCA1* positively regulates *PRR7* and *PRR9* expression, which in turn affect feedback regulation of *CCA1* (Farré et al., 2005). CCA1 can also interact with TOC1 to bind the *GI* promoter (Hsu and Harmer, 2014). *LUX* is a MYB transcript factor required for normal circadian rhythm that can repress *PRR9* expression (Chow et al., 2012). FKF1 is an F-box protein that forms a complex with GI to regulate the transition to flowering (Sawa et al., 2007). In rice, overexpression of *CCA1* or *GI* delays flowering (Hayama et al., 2003). The presence of these important circadian genes in our DEGs suggests that they may also play a critical role in anther development in response to photoperiod and warrant further investigation as potential candidates for PGMS line creation.

### Protein Metabolism and Phytohormones Play a Key Role in Photoperiod-Sensitive Anther Development

Weighted gene co-expression network analysis (WGCNA), which evaluates potential interaction between all expressed genes, revealed large gene clusters and regulatory networks accociated with photoperiod length and confirmed that gene expression differs significantly between SD and LD anthers. Four modules were identified with a high correlation to photoperiod: genes in turquoise (5852 genes) and magenta (1132 genes) modules were up-regulated in LD anthers, while genes in blue (4929 genes) and yellow (2698 genes) modules were up-regulated in SD anthers (Figure 4 and Supplementary Figure 8).

These modules were generally enriched with the same GO terms as DEGs (Figures 2, 5), but the blue module was also enriched in genes from new categories, i.e., protein-related and macromolecule-related processes, which contained *TMS10* (thermosensitive male sterility 10), *TMS10L* (thermosensitive male sterility 10-like), and *UbL404* (ubiquitin fusion ribosomal protein L40) genes. *TMS10* and *TMS10L* are two thermosensitive genes that redundantly regulate post-meiotic tapetal development and pollen development in low and high temperatures (Yu et al., 2017). UbL404 is a target of *TMS5*, which encodes a conserved RNase Z protein. UbL404 is degraded by RNase Z in wild-type plants, but accumulates in *tms5* plants at high temperatures, causing temperature-sensitive male sterility (Zhou et al., 2014). A subsequent mutation in *GATA10*, which decreases UbL404 levels, can restore fertility at high temperatures (Jin et al., 2019). Our modules thus contain known PGMS and TGMS effectors and are likely to be a good source to uncover as-yet-unknown genes affecting PGMS.

The “protein process” GO category also contained genes associated with light response and anther-specific genes (Figure 5 and Supplementary Data Sheet 7). Light response genes included *PHYB*, *PHYC*, and *Hd6*. PHYB and PHYC are photoreceptors in rice, single mutants of which induce early flowering; a triple mutant with PHYA causes incomplete sterility (Takano et al., 2005). *Hd6* encode a casein kinase II that delays heading time in LD conditions (Takahashi et al., 2001). Anther-specific genes included *Pollen Mitosis Relative (PMR)*, essential for correct pollen mitosis (Liu et al., 2017), and *DWARF AND RUNTISH SPIKELET (DRUS1)* and *DRUS2*, which regulate anther development and pollen maturation redundantly through sugar utilization or conversion (Pu et al., 2017). Genes involved in the MAPK (mitogen-activated protein kinase) and Ca^2+^-related signaling pathway were also found, such as *Calcium-dependent protein kinase 2* (*OsCDPK2*), which is repressed by light and disrupts seed development (Frattini et al., 1999; Morello et al., 2000). Genes associated with phytohormone signaling included brassinosteroid-signaling kinase (*BSK*) family members and *Ethylene Receptor 2* (*ETR2*). Thus, while mitosis and sugar transport are key biological processes occurring in stage 11 anthers, hormone, and other signaling pathways are also very active during anther late development.

Our WGCNA analysis revealed 496 hub genes in these four modules (Supplementary Data Sheet 6). A known PGMS gene, *CSA*, and TGMS gene, *Ugp1*, were among these hub genes, both known to affect male sterility through carbohydrate metabolism (Chen et al., 2007; Zhang et al., 2013). Other genes affecting carbohydrate metabolism were also among the hub genes, including *CAP1*, *DPE2*, and *OsSWN1*. These results provide further evidence that carbohydrate metabolism is a key characteristic of late anther development affected by photoperiod. Multiple phytohormone-related genes were also present in the hubs: *ABA 8’-Hydroxylase gene 3* (*OsABA8ox3*) is a key gene in abscisic acid (ABA) catabolism (Zhu et al., 2009); *OsNAP* can be induced by ABA and, in turn, affects ABA biosynthesis (Liang et al., 2014); *Gibberellin 20-oxidase 3* (*OsGA20ox3*) and *Gibberellin 2-oxidase 4* (*OsGA2ox4*) affect gibberellic acid biosynthesis, *GA20ox3*, in a temperature-dependent manner (Lo et al., 2008; Sakata et al., 2014); *Dioxygenase of auxin oxidation* (*DAO*) affects the auxin pathway (Zhao et al., 2013); and *SK3/SHAGGY-like kinase* (*GSK2*) acts as a negative regulator of brassinosteroid signaling (Tong et al., 2012). Phytohormones thus appear to be key regulators mediating the adaption of anther development to changing day length.

### TGMS and PGMS Share Regulatory Network Elements

Given the presence of *CSA* and *Ugp1* as hub genes, we searched for, and found, other hub genes regulating environment-sensitive male sterility (Table 1 and Supplementary Data Sheet 6). *TMS5* was clustered in the red module, which did not show significant correlation with photoperiod. *OsPDCD5*, *Ugp2*, and *Osgata10* clustered in the turquoise module, while *TMS10* clustered in the blue module. Both *OsPDCD5* and *TMS10* are involved in PCD, which, in rice anthers, begins before stage 11; had younger anthers been used in our analysis, WGCNA significance scores for these genes may have been higher.

Analysis of interaction networks of key hub genes revealed further interactions between PGMS gene *CSA* and TGMS gene *Ugp1* that two genes share large part of co-expressed genes. Although these genes were up- and down-regulated in an opposite pattern *CSA* and *Ugp1*, these genes are likely to be common targets of *Ugp1* and *CSA* regulation and have similar roles in co-regulating photoperiod- or temperature-sensitive anther development. This common list included genes encoding zinc finger, MADS-Box, and MYB transcript factors, which may play a key relational role in mediating photoperiod-associated male reproduction. Moreover, carbohydrate metabolic genes and transport genes like invertase were also detected, and these genes may be associated with the sugar transport and metabolism under SD and LD. Further analysis on 38 hub genes highly co-expressed with *CSA* and *Ugp1* may reveal key genes regulating photoperiod-dependent male fertility.

In summary, we provided a transcriptomic view in anther development at different photoperiods and narrowed down candidates not only for future discovery of new PGMS but also for genes that interacted with reported EGMS, *CSA* and *Ugp1*, based on DEG analysis and WGCNA. Further investigations to confirm the biological function of these genes will reveal new genetic and molecular controls of PGMS in rice and possible other crops.

## Data Availability Statement

The datasets presented in this study can be found in online repositories. The names of the repository/repositories and accession number(s) can be found below: https://www.ncbi.nlm.nih.gov/, GSE163030.

## Author Contributions

DZ designed the study. DW, JL, YL, and SS performed experiments. SS analyzed the RNA-seq data and drafted the manuscript. WC, GL, XZ, and DZ revised the manuscript. All authors contributed to the article and approved the submitted version.

## Conflict of Interest

The authors declare that the research was conducted in the absence of any commercial or financial relationships that could be construed as a potential conflict of interest.

## Footnotes

1 http://rice.plantbiology.msu.edu/pub/data/Eukaryotic_Projects/o_sativa/annotation_dbs/pseudomolecules/version_7.0/all.dir/

2 http://systemsbiology.cau.edu.cn/agriGOv2/

## References

[B1] Adrian D. (2019). *venn: Draw Venn Diagrams. R Package Version 1.8.* Available online at: https://Cran.R-project.org/package=venn (Accessed May 25, 2019).

[B2] Akdogan G. Kubota J. Kubo A. Takaha T. Kitamura S. (2011). Expression and characterization of rice disproportionating enzymes. *J. Appl. Glycosci.* 58 99–105. 10.5458/jag.jag.JAG-2010_026

[B3] Audic S. Claverie J. M. (1997). The significance of digital gene expression profiles. *Genome Res.* 7 986–995. 10.1101/gr.7.10.986 9331369

[B4] Bañuelos M. A. A. Garciadeblas B. Cubero B. Rodrıìguez-Navarro A. (2002). Inventory and functional characterization of the HAK potassium transporters of Rice. *Plant Physiol.* 130 784–795. 10.1104/pp.007781 12376644 PMC166606

[B5] Chai M. Bellizzi M. Wan C. Cui Z. Li Y. Wang G.-L. (2015). The NAC transcription factor OsSWN1 regulates secondary cell wall development in *Oryza sativa*. *J. Plant Biol.* 58 44–51. 10.1007/s12374-014-0400-y

[B6] Chen H. Zhang Z. Ni E. Lin J. Peng G. Huang J. (2020). HMS1 interacts with HMS1I to regulate very-long-chain fatty acid biosynthesis and the humidity-sensitive genic male sterility in rice (Oryza sativa). *New Phytol.* 225 2077–2093. 10.1111/nph.16288 31663135

[B7] Chen L.-Y. Xiao Y.-H. Lei D.-Y. (2010). Mechanism of sterility and breeding strategies for photoperiod/thermo-sensitive genic male sterile rice. *Rice Sci.* 17 161–167. 10.1016/s1672-6308(09)60012-3

[B8] Chen R. Zhao X. Shao Z. Wei Z. Wang Y. Zhu L. (2007). Rice UDP-glucose pyrophosphorylase1 is essential for pollen callose deposition and its cosuppression results in a new type of thermosensitive genic male sterility. *Plant Cell* 19 847–861. 10.1105/tpc.106.044123 17400897 PMC1867369

[B9] Chen Y. Ma J. Miller A. J. Luo B. Wang M. Zhu Z. (2016). OsCHX14 is involved in the K+ homeostasis in rice (*Oryza sativa*) flowers. *Plant Cell Physiol.* 57 1530–1543. 10.1093/pcp/pcw088 27903806

[B10] Cheng S. H. Zhuang J. Y. Fan Y. Y. Du J. H. Cao L. Y. (2007). Progress in research and development on hybrid rice: a super-domesticate in China. *Ann. Bot.* 100 959–966. 10.1093/aob/mcm121 17704538 PMC2759200

[B11] Cho J. I. Ryoo N. Eom J. S. Lee D. W. Kim H. B. Jeong S. W. (2009). Role of the rice hexokinases OsHXK5 and OsHXK6 as glucose sensors. *Plant Physiol.* 149 745–759. 10.1104/pp.108.131227 19010999 PMC2633841

[B12] Cho J.-I. Ryoo N. Ko S. Lee S.-K. Lee J. Jung K.-H. (2006). Structure, expression, and functional analysis of the hexokinase gene family in rice (*Oryza sativa* L.). *Planta* 224 598–611. 10.1007/s00425-006-0251-y 16552590

[B13] Chow B. Y. Helfer A. Nusinow D. A. Kay S. A. (2012). ELF3 recruitment to the PRR9 promoter requires other evening complex members in the *Arabidopsis* circadian clock. *Plant Signaling Behav.* 7 170–173. 10.4161/psb.18766 22307044 PMC3405715

[B14] Deng W. Li R. Xu Y. Mao R. Chen S. Chen L. (2020). A lipid transfer protein variant with a mutant eight-cysteine motif causes photoperiod- and thermo-sensitive dwarfism in rice. *J. Exp. Bot.* 71 1294–1305. 10.1093/jxb/erz500 31701134 PMC7031082

[B15] Deng X. Han X. Yu S. Liu Z. Guo D. He Y. (2020). OsINV3 and its homolog, OsINV2, control grain size in rice. *Int J Mol Sci* 21:2199. 10.3390/ijms21062199 32209971 PMC7139340

[B16] Ding J. Lu Q. Ouyang Y. Mao H. Zhang P. Yao J. (2012a). A long noncoding RNA regulates photoperiod-sensitive male sterility, an essential component of hybrid rice. *Proc. Natl. Acad. Sci. U.S.A.* 109 2654–2659. 22308482 10.1073/pnas.1121374109 PMC3289353

[B17] Ding J. Shen J. Mao H. Xie W. Li X. Zhang Q. (2012b). RNA-directed DNA methylation is involved in regulating photoperiod-sensitive male sterility in rice. *Mol. Plant* 5 1210–1216. 10.1093/mp/sss095 23024213

[B18] Fan Y. Yang J. Mathioni S. M. Yu J. Shen J. Yang X. (2016). PMS1T, producing phased small-interfering RNAs regulates photoperiod-sensitive male sterility in rice. *Proc. Natl. Acad. Sci. U.S.A.* 113 15144–15149. 27965387 10.1073/pnas.1619159114 PMC5206514

[B19] Farré E. M. Harmer S. L. Harmon F. G. Yanovsky M. J. Kay S. A. (2005). Overlapping and distinct roles of PRR7 and PRR9 in the *Arabidopsis* circadian clock. *Curr. Biol.* 15 47–54. 10.1016/j.cub.2004.12.067 15649364

[B20] Frattini M. Morello L. Breviario D. (1999). Rice calcium-dependent protein kinase isoforms OsCDPK2 and OsCDPK11 show different responses to light and different expression patterns during seed development. *Plant Mol. Biol.* 41 753–764. 10.1023/A:1006316422400 10737140

[B21] Hayama R. Yokoi S. Tamaki S. Yano M. Shimamoto K. (2003). Adaptation of photoperiodic control pathways produces short-day flowering in rice. *Nature* 422 719–722. 10.1038/nature01549 12700762

[B22] Hirose T. Zhang Z. Miyao A. Hirochika H. Ohsugi R. Terao T. (2010). Disruption of a gene for rice sucrose transporter, OsSUT1, impairs pollen function but pollen maturation is unaffected. *J. Exp. Bot.* 61 3639–3646. 10.1093/jxb/erq175 20603282 PMC2921200

[B23] Horvath S. Dong J. (2008). Geometric interpretation of gene coexpression network analysis. *PLoS Comput Biol.* 4:e1000117. 10.1371/journal.pcbi.1000117 18704157 PMC2446438

[B24] Hsu P. Y. Harmer S. L. (2014). Wheels within wheels: the plant circadian system. *Trends Plant Sci.* 19 240–249. 10.1016/j.tplants.2013.11.007 24373845 PMC3976767

[B25] Hwang S.-K. Koper K. Satoh H. Okita T. W. (2016). Rice endosperm starch phosphorylase (Pho1) assembles with disproportionating enzyme (Dpe1) to form a protein complex that enhances synthesis of malto-oligosaccharides. *J. Biol. Chem.* 291 19994–20007. 27502283 10.1074/jbc.M116.735449 PMC5025686

[B26] Izawa T. Oikawa T. Sugiyama N. Tanisaka T. Yano M. Shimamoto K. (2002). Phytochrome mediates the external light signal to repress FT orthologs in photoperiodic flowering of rice. *Genes Dev.* 16 2006–2020. 12154129 10.1101/gad.999202 PMC186415

[B27] Jabnoune M. Espeout S. Mieulet D. Fizames C. Verdeil J.-L. Conéjéro G. (2009). Diversity in expression patterns and functional properties in the rice HKT transporter family. *Plant Physiol.* 150 1955–1971. 10.1104/pp.109.138008 19482918 PMC2719131

[B28] Jin J. Gui S. Li Q. Wang Y. Zhang H. Zhu Z. (2019). The transcription factor GATA10 regulates fertility conversion of a two-line hybrid tms5 mutant rice via the modulation of UbL40 expression. *J. Integr. Plant Biol.* 62 1034–1056. 10.1111/jipb.12871 31486580 PMC7383616

[B29] Jung K. H. Han M. J. Lee D. Y. Lee Y. S. Schreiber L. Franke R. (2006). Wax-deficient anther1 is involved in cuticle and wax production in rice anther walls and is required for pollen development. *Plant Cell* 18 3015–3032. 10.1105/tpc.106.042044 17138699 PMC1693940

[B30] Kanehisa M. Goto S. (2000). KEGG: kyoto encyclopedia of genes and genomes. *Nucleic Acids Res.* 28 27–30. 10592173 10.1093/nar/28.1.27 PMC102409

[B31] Kawahara Y. de la Bastide M. Hamilton J. P. Kanamori H. McCombie W. R. Ouyang S. (2013). Improvement of the *Oryza sativa* nipponbare reference genome using next generation sequence and optical map data. *Rice* 6:4. 10.1186/1939-8433-6-4 24280374 PMC5395016

[B32] Khush G. S. (2001). Green revolution: the way forward. *Nat. Rev. Genet.* 2 815–822. 10.1038/35093585 11584298

[B33] Kim H.-B. Cho J.-I. Ryoo N. Shin D.-H. Park Y.-I. Hwang Y.-S. (2016). Role of rice cytosolic hexokinase OsHXK7 in sugar signaling and metabolism. *J. Integr. Plant Biol.* 58 127–135. 10.1111/jipb.12366 25951042

[B34] Kim Y.-J. Zhang D. (2018). Molecular control of male fertility for crop hybrid breeding. *Trends Plant Sci.* 23 53–65. 10.1016/j.tplants.2017.10.001 29126789

[B35] Langfelder P. Horvath S. (2008). WGCNA: an R package for weighted correlation network analysis. *BMC Bioinf.* 9:559. 10.1186/1471-2105-9-559 19114008 PMC2631488

[B36] Liang C. Wang Y. Zhu Y. Tang J. Hu B. Liu L. (2014). OsNAP connects abscisic acid and leaf senescence by fine-tuning abscisic acid biosynthesis and directly targeting senescence-associated genes in rice. *Proc. Natl. Acad. Sci. U.S.A.* 111 10013–10018. 10.1073/pnas.1321568111 24951508 PMC4103337

[B37] Lim J. D. Cho J.-I. Park Y.-I. Hahn T.-R. Choi S.-B. Jeon J.-S. (2006). Sucrose transport from source to sink seeds in rice. *Physiol. Plant.* 126 572–584. 10.1111/j.1399-3054.2006.00654.x

[B38] Liu L. Zheng C. Kuang B. Wei L. Yan L. Wang T. (2016). Receptor-like kinase RUPO interacts with potassium transporters to regulate pollen tube growth and integrity in rice. *PLoS Genet.* 12:e1006085. 10.1371/journal.pgen.1006085 27447945 PMC4957769

[B39] Liu Y. Xu Y. Ling S. Liu S. Yao J. (2017). Anther-preferential expressing gene PMR is essential for the mitosis of pollen development in rice. *Plant Cell Rep.* 36 919–931. 10.1007/s00299-017-2123-2 28299429

[B40] Livak K. J. Schmittgen T. D. (2001). Analysis of relative gene expression data using real-time quantitative PCR and the 2^−ΔΔ^CT method. *Methods* 25 402–408. 10.1006/meth.2001.1262 11846609

[B41] Lo S.-F. Yang S.-Y. Chen K.-T. Hsing Y.-I. Zeevaart J. A. D. Chen L.-J. (2008). A novel class of gibberellin 2-oxidases control semidwarfism, tillering, and root development in rice. *Plant Cell* 20 2603–2618. 10.1105/tpc.108.060913 18952778 PMC2590730

[B42] Lu K. Li T. He J. Chang W. Zhang R. Liu M. (2017). qPrimerDB: a thermodynamics-based gene-specific qPCR primer database for 147 organisms. *Nucleic Acids Res.* 46 D1229–D1236. 10.1093/nar/gkx725 28977518 PMC5753361

[B43] Michel D. Jérôme S. Richard C. Christophe S. Farid R. Pierre L. (2001). Rice genomics: present and future. *Plant Physiol. Biochem.* 39 323–334.

[B44] Morello L. Frattini M. Gianì S. Christou P. Breviario D. (2000). Overexpression of The calcium-dependent protein kinase OsCDPK2 in transgenic rice is repressed by light in leaves and disrupts seed development. *Transgenic Res.* 9 453–462. 10.1023/A:1026555021606 11206974

[B45] Mou T. (2016). The research progress and prospects of two-line hybrid rice in China. *Chin. Sci. Bull.* 61 3761–3769. 10.1360/n972016-01045

[B46] Mu H. Ke J. Liu W. Zhuang C. Yip W. (2009). UDP-glucose pyrophosphorylase2 (OsUgp2), a pollen-preferential gene in rice, plays a critical role in starch accumulation during pollen maturation. *Sci. Bull.* 54 234–243. 10.1007/s11434-008-0568-y

[B47] Murakami M. Ashikari M. Miura K. Yamashino T. Mizuno T. (2003). The evolutionarily conserved OsPRR quintet: rice pseudo-response regulators implicated in circadian rhythm. *Plant Cell Physiol.* 44 1229–1236. 10.1093/pcp/pcg135 14634161

[B48] Murakami M. Matsushika A. Ashikari M. Yamashino T. Mizuno T. (2005). Circadian-associated rice pseudo response regulators (OsPRRs): insight into the control of flowering time. *Biosci. Biotechnol. Biochem.* 69 410–414. 10.1271/bbb.69.410 15725670

[B49] Murakami M. Tago Y. Yamashino T. Mizuno T. (2007). comparative overviews of clock-associated genes of *Arabidopsis thaliana* and *Oryza sativa*. *Plant Cell Physiol.* 48 110–121. 10.1093/pcp/pcl043 17132630

[B50] Nakagawa Y. Hanaoka H. Kobayashi M. Miyoshi K. Miwa K. Fujiwara T. (2007). Cell-type specificity of the expression of Os BOR1, a rice efflux boron transporter gene, is regulated in response to boron availability for efficient boron uptake and xylem loading. *Plant Cell* 19 2624–2635. 10.1105/tpc.106.049015 17675406 PMC2002629

[B51] Ngampanya B. Sobolewska A. Takeda T. Toyofuku K. Narangajavana J. Ikeda A. (2003). Characterization of rice functional monosaccharide transporter, OsMST5. *Biosci. Biotechnol. Biochem.* 67 556–562. 10.1271/bbb.67.556 12723603

[B52] Oliver S. N. Van Dongen J. T. Alfred S. C. Mamun E. A. Zhao X. Saini H. S. (2005). Cold-induced repression of the rice anther-specific cell wall invertase gene OSINV4 is correlated with sucrose accumulation and pollen sterility. *PlantCell Environ.* 28 1534–1551.

[B53] Pu C.-X. Han Y.-F. Zhu S. Song F.-Y. Zhao Y. Wang C.-Y. (2017). The rice receptor-like kinases dwarf and runtish spikelet1 and 2 repress cell death and affect sugar utilization during reproductive development. *Plant Cell* 29 70–89. 10.1105/tpc.16.00218 28082384 PMC5304344

[B54] Raivo Kolde (2019). *pheatmap: Pretty Heatmaps. R package Version 1.0.12.* Available online at: https://CRAN.R-project.org/package=pheatmap (accessed January 04, 2019).

[B55] Rodríguez-Navarro A. Rubio F. (2006). High-affinity potassium and sodium transport systems in plants. *J. Exp. Bot.* 57 1149–1160. 10.1093/jxb/erj068 16449373

[B56] Sakata T. Oda S. Tsunaga Y. Shomura H. Kawagishi-Kobayashi M. Aya K. (2014). Reduction of gibberellin by low temperature disrupts pollen development in rice. *Plant Physiol.* 164 2011–2019. 10.1104/pp.113.234401 24569847 PMC3982758

[B57] Sato Y. Takehisa H. Kamatsuki K. Minami H. Namiki N. Ikawa H. (2012). RiceXPro version 3.0: expanding the informatics resource for rice transcriptome. *Nucleic Acids Res.* 41 D1206–D1213. 10.1093/nar/gks1125 23180765 PMC3531122

[B58] Sawa M. Nusinow D. A. Kay S. A. Imaizumi T. (2007). FKF1 and GIGANTEA complex formation is required for day-length measurement in *Arabidopsis*. *Science* 318:261. 10.1126/science.1146994 17872410 PMC3709017

[B59] Shannon P. Markiel A. Ozier O. Baliga N. S. Wang J. T. Ramage D. (2003). Cytoscape: a software environment for integrated models of biomolecular interaction networks. *Genome Res.* 13 2498–2504. 14597658 10.1101/gr.1239303 PMC403769

[B60] Shi M. S. (1981). Breeding and using of a two-usage natural mutant in late japonica rice (in Chinese). *Hubei. Agric. Sci.* 7 1–3.

[B61] Shi X. Sun X. Zhang Z. Feng D. Zhang Q. Han L. (2015). Glucan synthase-like 5 (GSL5) plays an essential role in male fertility by regulating callose metabolism during microsporogenesis in rice. *Plant Cell Physiol.* 56 497–509. 10.1093/pcp/pcu193 25520407

[B62] Sugiyama N. Izawa T. Oikawa T. Shimamoto K. (2001). Light regulation of circadian clock-controlled gene expression in rice. *Plant J.* 26 607–615. 10.1046/j.1365-313x.2001.01063.x 11489174

[B63] Suzuki K. Yamaji N. Costa A. Okuma E. Kobayashi N. I. Kashiwagi T. (2016). OsHKT1;4-mediated Na+ transport in stems contributes to Na+ exclusion from leaf blades of rice at the reproductive growth stage upon salt stress. *BMC Plant Biol.* 16:22. 10.1186/s12870-016-0709-4 26786707 PMC4719677

[B64] Takahashi Y. Shomura A. Sasaki T. Yano M. (2001). Hd6, a rice quantitative trait locus involved in photoperiod sensitivity, encodes the α subunit of protein kinase CK2. *Proc. Natl. Acad. Sci. U.S.A.* 98 7922–7927. 10.1073/pnas.111136798 11416158 PMC35444

[B65] Takano M. Inagaki N. Xie X. Yuzurihara N. Hihara F. Ishizuka T. (2005). Distinct and cooperative functions of phytochromes A, B, and C in the control of deetiolation and flowering in rice. *Plant Cell* 17 3311–3325. 10.1105/tpc.105.035899 16278346 PMC1315371

[B66] Tanaka K. Murata K. Yamazaki M. Onosato K. Miyao A. Hirochika H. (2003). Three distinct rice cellulose synthase catalytic subunit genes required for cellulose synthesis in the secondary wall. *Plant Physiol.* 133 73–83. 10.1104/pp.103.022442 12970476 PMC196581

[B67] Tanaka N. Uraguchi S. Saito A. Kajikawa M. Kasai K. Sato Y. (2013). Roles of pollen-specific boron efflux transporter, OsBOR4, in the rice fertilization process. *Plant Cell Physiol.* 54 2011–2019. 10.1093/pcp/pct136 24068795

[B68] Tian T. Liu Y. Yan H. You Q. Yi X. Du Z. (2017). agriGO v2.0: a GO analysis toolkit for the agricultural community, 2017 update. *Nucleic Acids Res.* 45 W122–W129. 10.1093/nar/gkx382 28472432 PMC5793732

[B69] Tong H. Liu L. Jin Y. Du L. Yin Y. Qian Q. (2012). Dwarf and low-tillering acts as a direct downstream target of a GSK3/SHAGGY-like kinase to mediate brassinosteroid responses in rice. *Plant Cell* 24 2562–2577. 10.1105/tpc.112.097394 22685166 PMC3406904

[B70] Ueda K. Yoshimura F. Miyao A. Hirochika H. Nonomura K. Wabiko H. (2013). Collapsed abnormal pollen1 gene encoding the Arabinokinase-like protein is involved in pollen development in rice. *Plant Physiol.* 162 858–871. 10.1104/pp.113.216523 23629836 PMC3668075

[B71] Wan L. Zha W. Cheng X. Liu C. Lv L. Liu C. (2011). A rice beta-1,3-glucanase gene Osg1 is required for callose degradation in pollen development. *Planta* 233 309–323. 10.1007/s00425-010-1301-z 21046148

[B72] Wang Y. Zha X. Zhang S. Qian X. Dong X. Sun F. (2010). Down-regulation of the OsPDCD5 gene induced photoperiod-sensitive male sterility in rice. *Plant Sci.* 178 221–228. 10.1016/j.plantsci.2009.12.001

[B73] Wang K. Singh D. Zeng Z. Coleman S. J. Huang Y. Savich G. L. (2010). MapSplice: accurate mapping of RNA-seq reads for splice junction discovery. *Nucleic Acids Res.* 38:e178. 10.1093/nar/gkq622 20802226 PMC2952873

[B74] Wang Y. Xu H. Wei X. Chai C. Xiao Y. Zhang Y. (2007). Molecular cloning and expression analysis of a monosaccharide transporter gene OsMST4 from rice (*Oryza sativa* L.). *Plant Mol. Biol.* 65 439–451. 10.1007/s11103-007-9228-x 17874189

[B75] Wei S. Hu W. Deng X. Zhang Y. Liu X. Zhao X. (2014). A rice calcium-dependent protein kinase OsCPK9 positively regulates drought stress tolerance and spikelet fertility. *BMC Plant Biol.* 14:133. 10.1186/1471-2229-14-133 24884869 PMC4036088

[B76] Wickham H. (2016). *ggplot2: Elegant Graphics for Data Analysis*. Berlin: Springer.

[B77] Wu L. Guan Y. Wu Z. Yang K. Lv J. Converse R. (2014). OsABCG15 encodes a membrane protein that plays an important role in anther cuticle and pollen exine formation in rice. *Plant Cell Rep.* 33 1881–1899. 10.1007/s00299-014-1666-8 25138437 PMC4197380

[B78] Xu F. Q. Li X. R. Ruan Y. L. (2008). RNAi-mediated suppression of hexokinase gene OsHXK10 in rice leads to non-dehiscent anther and reduction of pollen germination. *Plant Sci.* 175 674–684. 10.1016/j.plantsci.2008.07.002

[B79] Xu Y. Liu S. Liu Y. Ling S. Chen C. Yao J. (2017). Hothead-like HTH1 is involved in anther cutin biosynthesis and is required for pollen fertility in rice. *Plant Cell Physiol.* 58 1238–1248. 10.1093/pcp/pcx063 28838125

[B80] Yamaji N. Takemoto Y. Miyaji T. Mitani-Ueno N. Yoshida K. T. Ma J. F. (2017). Reducing phosphorus accumulation in rice grains with an impaired transporter in the node. *Nature* 541 92–95. 10.1038/nature20610 28002408

[B81] Yao L. Zhang Y. Liu C. Liu Y. Wang Y. Liang D. (2018). OsMATL mutation induces haploid seed formation in indica rice. *Nat. Plants* 4 530–533. 10.1038/s41477-018-0193-y 29988153

[B82] Ye J. Huang M. Zhao S. Wu N. (2004). The relationship between the expression of osRACD and the fertility changes of photoperiod-sensitivegenic male sterile rice (in Chinese). *Prog. Nat. Sci* 14 166–172.

[B83] Yokosho K. Yamaji N. Ma J. F. (2016). OsFRDL1 expressed in nodes is required for distribution of iron to grains in rice. *J. Exp. Bot.* 67 5485–5494. 10.1093/jxb/erw314 27555544 PMC5049396

[B84] Yokosho K. Yamaji N. Ueno D. Mitani N. Ma J. F. (2009). OsFRDL1 is a citrate transporter required for efficient translocation of iron in rice. *Plant Physiol.* 149 297–305. 10.1104/pp.108.128132 19011004 PMC2613705

[B85] Yu G. Wang L.-G. Han Y. He Q.-Y. (2012). clusterProfiler: an R package for comparing biological themes among gene clusters. *OMICS* 16 284–287. 10.1089/omi.2011.0118 22455463 PMC3339379

[B86] Yu J. Han J. Kim Y. J. Song M. Yang Z. He Y. (2017). Two rice receptor-like kinases maintain male fertility under changing temperatures. *Proc. Natl. Acad. Sci.U.S.A.* 114 12327–12332. 10.1073/pnas.1705189114 29087306 PMC5699033

[B87] Yuan L. (1984). Hybrid rice in China. *Chin. J. Rice Sci.* 1 8–18.

[B88] Yuan L. (2004). Hybrid rice technology for food security in the world. *Crop Res.* 18 185–186.

[B89] Zhang D. Luo X. Zhu L. (2011). Cytological analysis and genetic control of rice anther development. *J. Genet. Genomics* 38 379–390. 10.1016/j.jgg.2011.08.001 21930097

[B90] Zhang H. Liang W. Yang X. Luo X. Jiang N. Ma H. (2010). Carbon starved anther encodes a MYB domain protein that regulates sugar partitioning required for rice pollen development. *Plant Cell* 22 672–689. 10.1105/tpc.109.073668 20305120 PMC2861464

[B91] Zhang H. Xu C. He Y. Zong J. Yang X. Si H. (2013). Mutation in CSA creates a new photoperiod-sensitive genic male sterile line applicable for hybrid rice seed production. *Proc. Natl. Acad. Sci. U.S.A.* 110 76–81. 23256151 10.1073/pnas.1213041110 PMC3538234

[B92] Zhang Q. Shen B. Z. Dai X. K. Mei M. H. Saghai Maroof M. A. Li Z. B. (1994). Using bulked extremes and recessive class to map genes for photoperiod-sensitive genic male sterility in rice. *Proc. Natl. Acad. Sci. U.S.A.* 91 8675–8679. 10.1073/pnas.91.18.8675 7915844 PMC44669

[B93] Zhao G. Shi J. Liang W. Xue F. Luo Q. Zhu L. (2015). Two ATP binding cassette G transporters, rice ATP binding cassette G26 and ATP binding cassette G15, collaboratively regulate rice male reproduction. *Plant Physiol.* 169 2064–2079. 10.1104/pp.15.00262 26392263 PMC4634043

[B94] Zhao Z. Zhang Y. Liu X. Zhang X. Liu S. Yu X. (2013). A role for a dioxygenase in auxin metabolism and reproductive development in rice. *Dev. Cell* 27 113–122. 10.1016/j.devcel.2013.09.005 24094741

[B95] Zhou H. Liu Q. Li J. Jiang D. Zhou L. Wu P. (2012). Photoperiod- and thermo-sensitive genic male sterility in rice are caused by a point mutation in a novel noncoding RNA that produces a small RNA. *Cell Res.* 22 649–660. 10.1038/cr.2012.28 22349461 PMC3317565

[B96] Zhou H. Zhou M. Yang Y. Li J. Zhu L. Jiang D. (2014). RNase ZS1 processes UbL40 mRNAs and controls thermosensitive genic male sterility in rice. *Nat. Commun.* 5:4884. 10.1038/ncomms5884 25208476

[B97] Zhou Y. F. Zhang X. Y. Xue Q. Z. (2011). Fine mapping and candidate gene prediction of the photoperiod and thermo-sensitive genic male sterile gene pms1(t) in rice. *J. Zhejiang Univ. Sci. B* 12 436–447. 10.1631/jzus.B1000306 21634036 PMC3109145

[B98] Zhu G. Ye N. Zhang J. (2009). Glucose-induced delay of seed germination in rice is mediated by the suppression of ABA catabolism rather than an enhancement of ABA biosynthesis. *Plant Cell Physiol.* 50 644–651. 10.1093/pcp/pcp022 19208695

[B99] Zou T. Xiao Q. Li W. Luo T. Yuan G. He Z. (2017). OsLAP6/OsPKS1, an orthologue of *Arabidopsis* PKSA/LAP6, is critical for proper pollen exine formation. *Rice* 10:53. 10.1186/s12284-017-0191-0 29282604 PMC5745217

