# Supplementary material for: Transcriptome Analysis Reveals Photoperiod-Associated Genes Expressed in Rice Anthers
Source: Front Plant Sci. 2021 Feb 26;12:621561. doi: 10.3389/fpls.2021.621561 (PMC7953911; doi:10.3389/fpls.2021.621561)
Supplement: Supplementary file 1 [file Image_1.pdf]

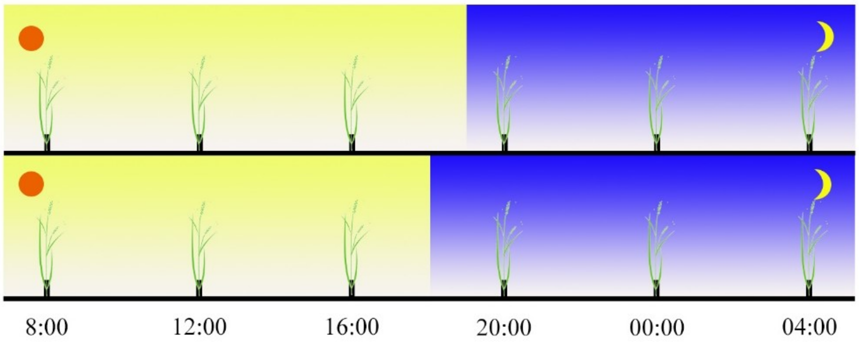

Supplementary Figure 1. The time point of collected samples.

The anther sample were collected every 4 hours from 8:00 to 04:00 under short-day(SD) and long-day(LD); 8:00, 12:00, 16:00 were under light condition and 20:00, 00:00, 04:00 were under dark condition.

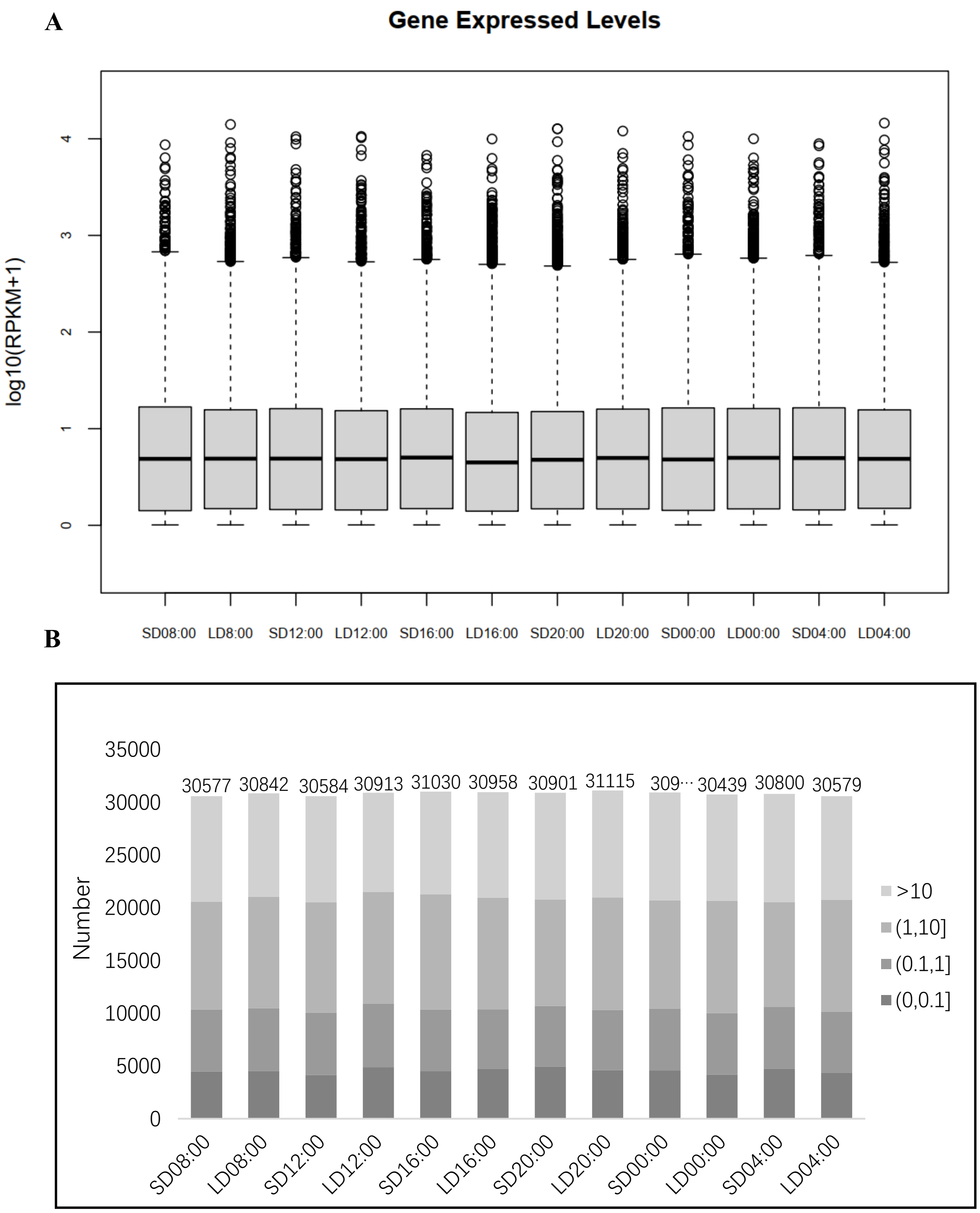

Supplementary Figure 2. Gene expression levels and number of expression genes (A)Box-plot of all gene expression levels base on RPKM, y-axis is log10(rpkm+1), x-axis represent different samples; (B)Bar-plot of number of expressed genes in each sample, y-axis is gene number, x-axis represent different samples, the diminishing gray shows different expressed levels based on RPKM.

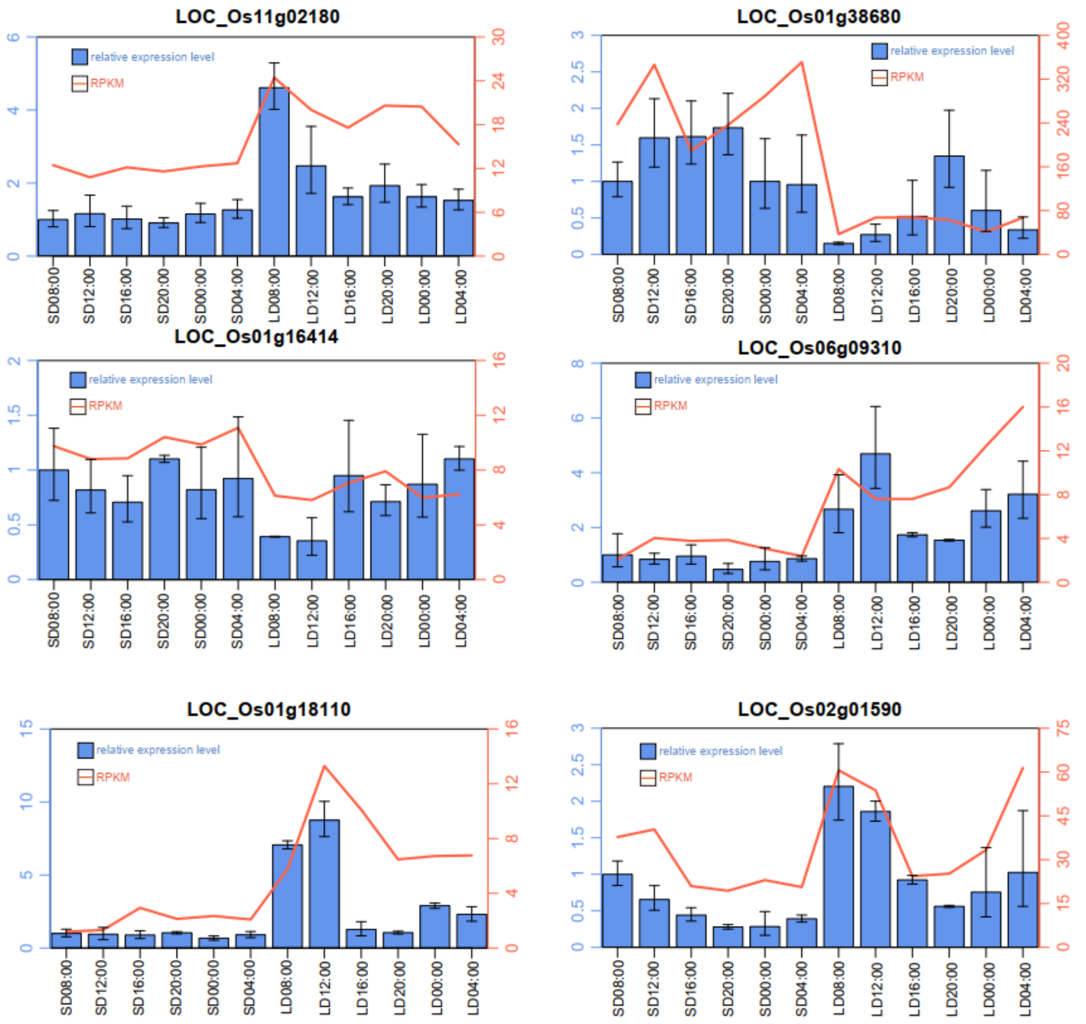

Supplementary Figure 3. Validation of the expression of RNA-seq data.
Gene expression levels were detected by qRT-PCR based on $2^{-\Delta\Delta Ct}$ method, represents the relative expression level and represents RPKM. The left Y-axis and the blue box are relative expression level, the right Y-axis and the orange line is RPKM.

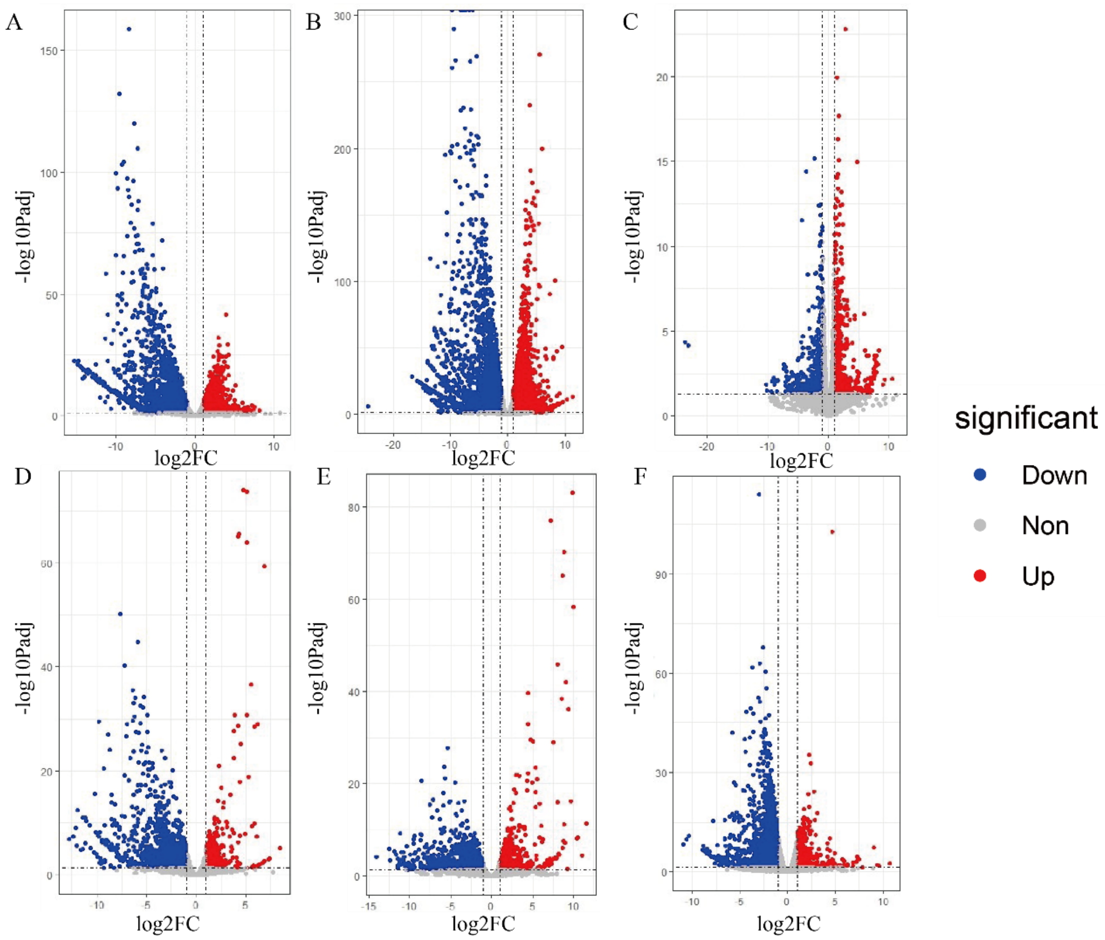

Supplementary Figure 4. Volcano plots of all time points' DEGs.

(A) 8:00. (B) 12:00. (C) 16:00. (D) 20:00. (E) 00:00. (F) 4:00. X-axis is log2 (fold change), Y-axis is -log10 (p.adjust), blue point means fold change of differential expression higher than 2 and p. adjust lower than 0.05.

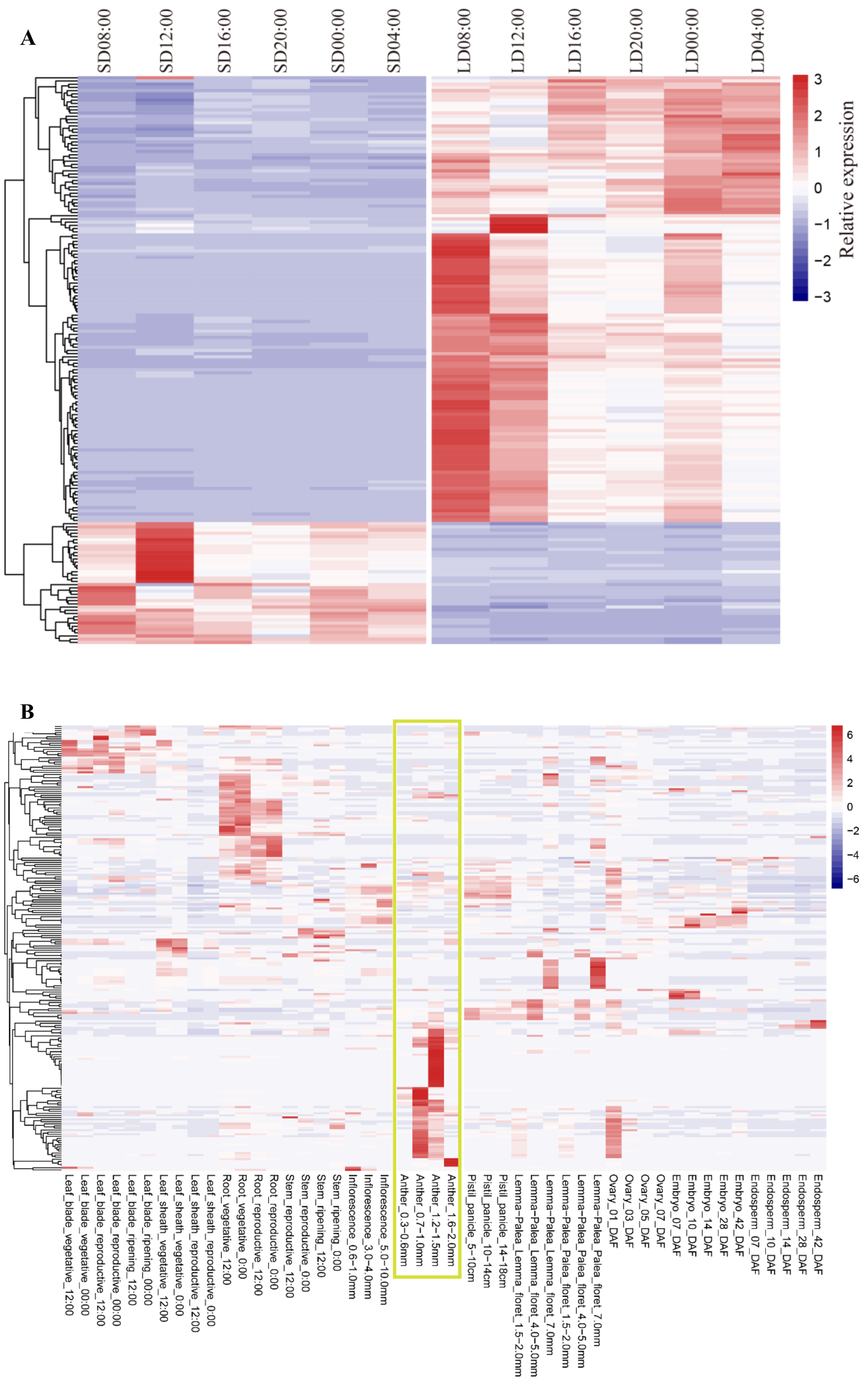
A
SD08:00
SD12:00
SD16:00
SD20:00
SD00:00
SD04:00
LD08:00
LD12:00
LD16:00
LD20:00
LD00:00
LD04:00
Relative expression
3
2
1
0
−1
−2
−3
B
6
4
2
0
−2
−4
−6
Leaf_blade_vegetative_12:00
Leaf_blade_vegetative_00:00
Leaf_blade_reproductive_12:00
Leaf_blade_reproductive_00:00
Leaf_blade_ripening_12:00
Leaf_blade_ripening_00:00
Leaf_sheath_vegetative_12:00
Leaf_sheath_vegetative_0:00
Leaf_sheath_reproductive_12:00
Leaf_sheath_reproductive_0:00
Root_vegetative_12:00
Root_vegetative_0:00
Root_reproductive_12:00
Root_reproductive_0:00
Stem_reproductive_12:00
Stem_reproductive_0:00
Stem_ripening_12:00
Stem_ripening_0:00
Inflorescence_0.6−1.0mm
Inflorescence_3.0−4.0mm
Inflorescence_5.0−10.0mm
Anther_0.3−0.6mm
Anther_0.7−1.0mm
Anther_1.2−1.5mm
Anther_1.6−2.0mm
Pistil_panicle_5−10cm
Pistil_panicle_10−14cm
Pistil_panicle_14−18cm
Lemma−Palea_Lemma_floret_1.5−2.0mm
Lemma−Palea_Lemma_floret_4.0−5.0mm
Lemma−Palea_Lemma_floret_7.0mm
Lemma−Palea_Palea_floret_1.5−2.0mm
Lemma−Palea_Palea_floret_4.0−5.0mm
Lemma−Palea_Palea_floret_7.0mm
Ovary_01_DAF
Ovary_03_DAF
Ovary_05_DAF
Ovary_07_DAF
Embryo_07_DAF
Embryo_10_DAF
Embryo_14_DAF
Embryo_28_DAF
Embryo_42_DAF
Endosperm_07_DAF
Endosperm_10_DAF
Endosperm_14_DAF
Endosperm_28_DAF
Endosperm_42_DAF

C

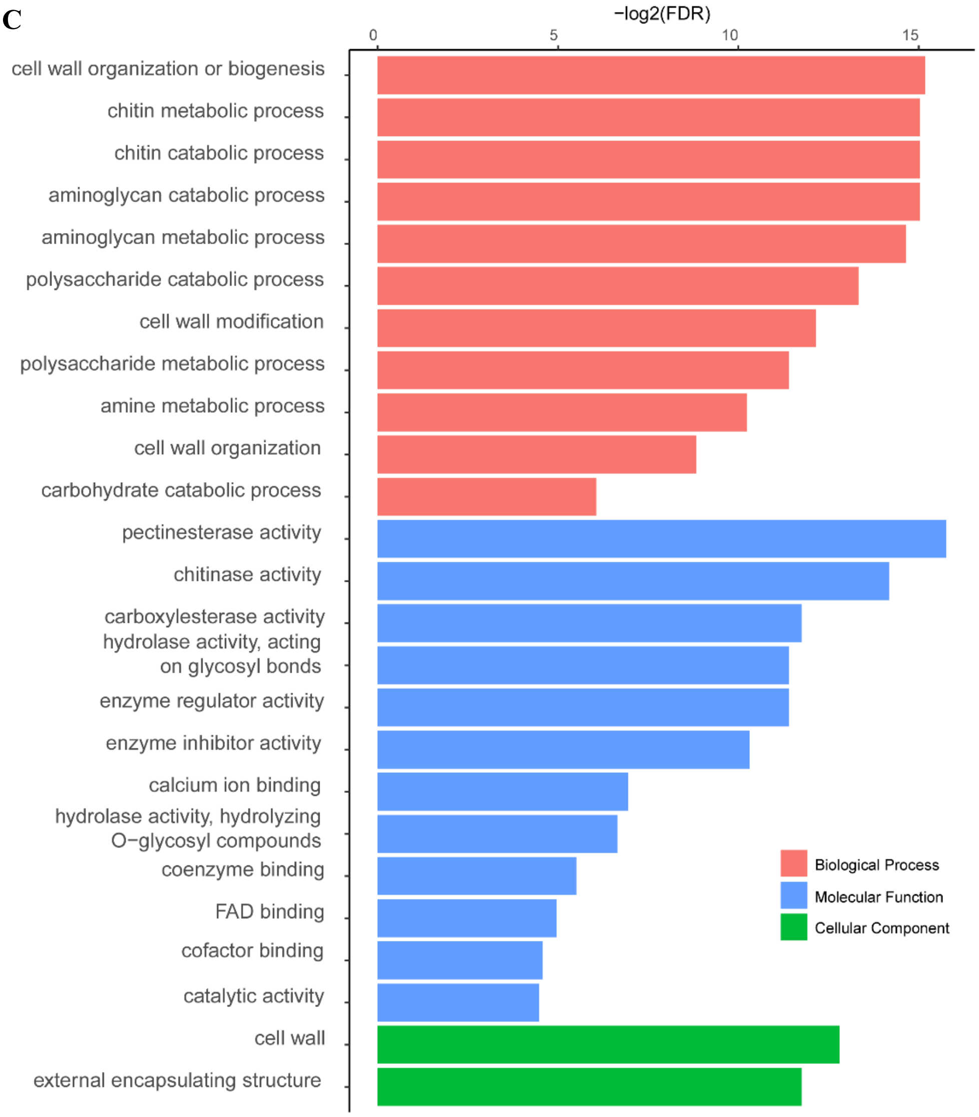

Supplementary Figure 5. DEGs of all time points.

(A) heatmap of 177 DEGs in anther; each row corresponds to a gene, each column corresponds to a time point, the color of every cell indicates the expression level based on z-score normalization. (B) heatmap of 177 DEGs in all tissues; each row corresponds to a gene, each column corresponds to a tissue. (C) significant GO enrichment of 177 DEGs; red bar is biological process, blue bar is molecular function, green bar is cellular component. X-axis is -log (FDR) and different GO terms is in Y-axis.

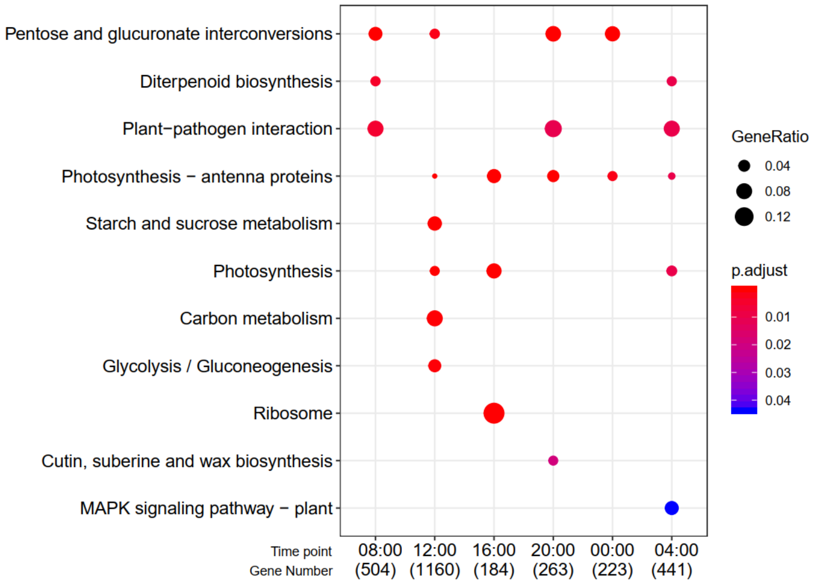

Supplementary Figure 6. KEGG enrichment analysis of all DEGs. X-axis is all time points, Y-axis is various KEGG terms, GeneRatio, depicted by circle size, indicates the rate of gene number of related term compared with all gene number in each time point; p.adjust, shown by color, indicates significance level of enrichment results from blue to red.

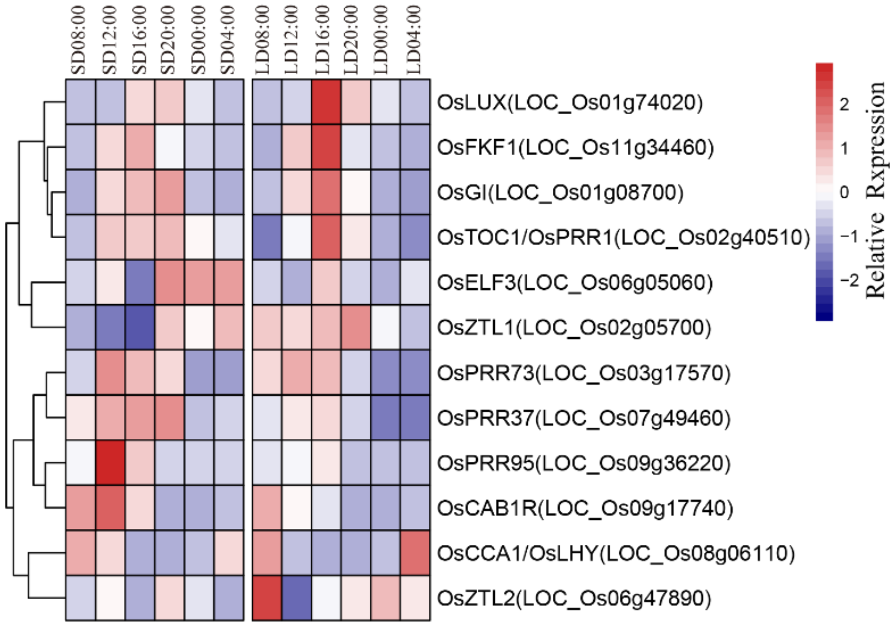

Supplementary Figure 7. Heatmaps and dendrograms of circadian rhythm genes. Blue color indicates the down-regulation in anthers; red color indicates up-regulation in anthers.

A

B

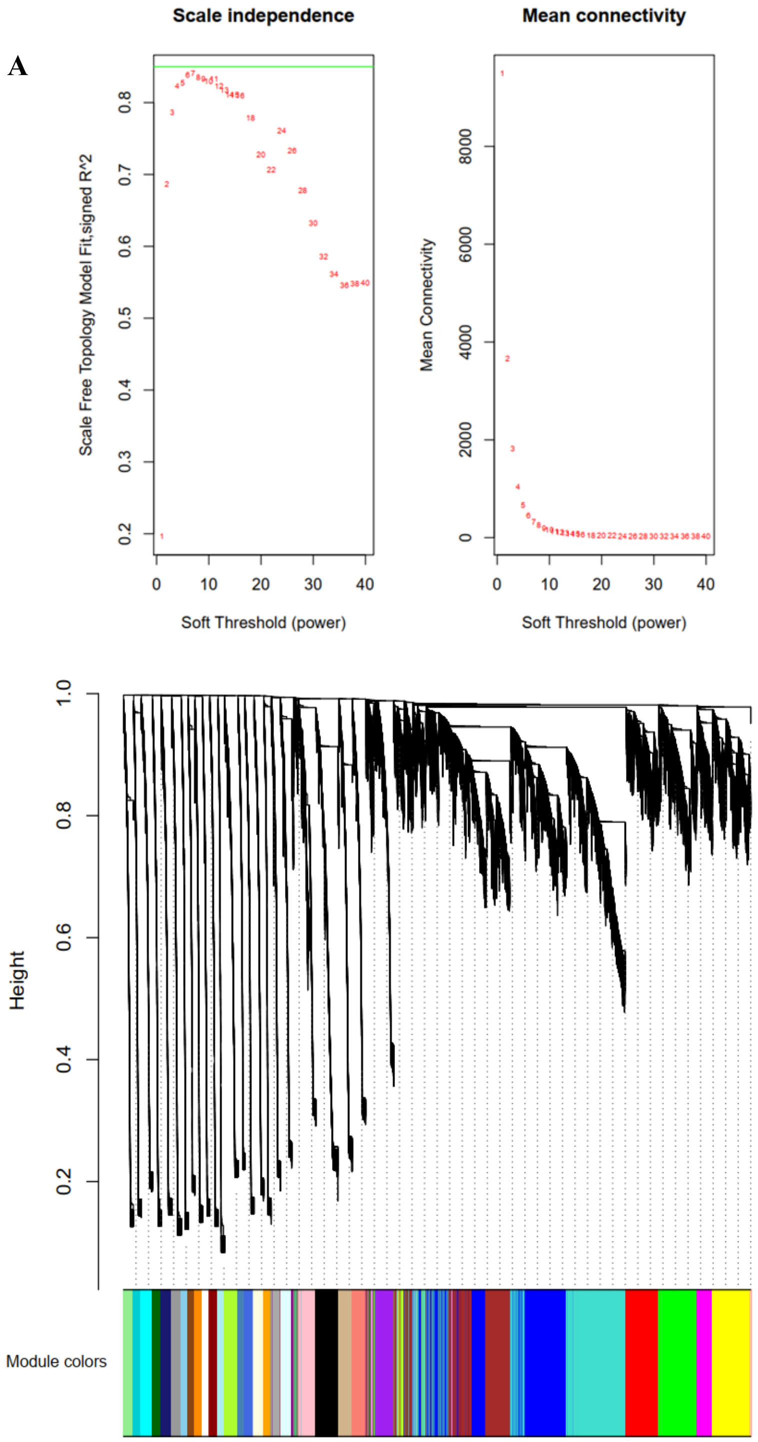

Supplementary Figure 8. (A) The network topology for various soft thresholding powers. The left panel shows the scale-free fit index (y-axis) as a function of the soft-thresholding power(x-axis). The right panel displays the mean connectivity (degree, y-axis) as a function of the soft-thresholding power(x-axis); (B) 35 modules of expressed genes were identified in all RNA-seq sets by clustering dendrograms analysis: each 'tree leaf' represents an independent gene; each 'branch' represents a module; the lowest panel show module colors.

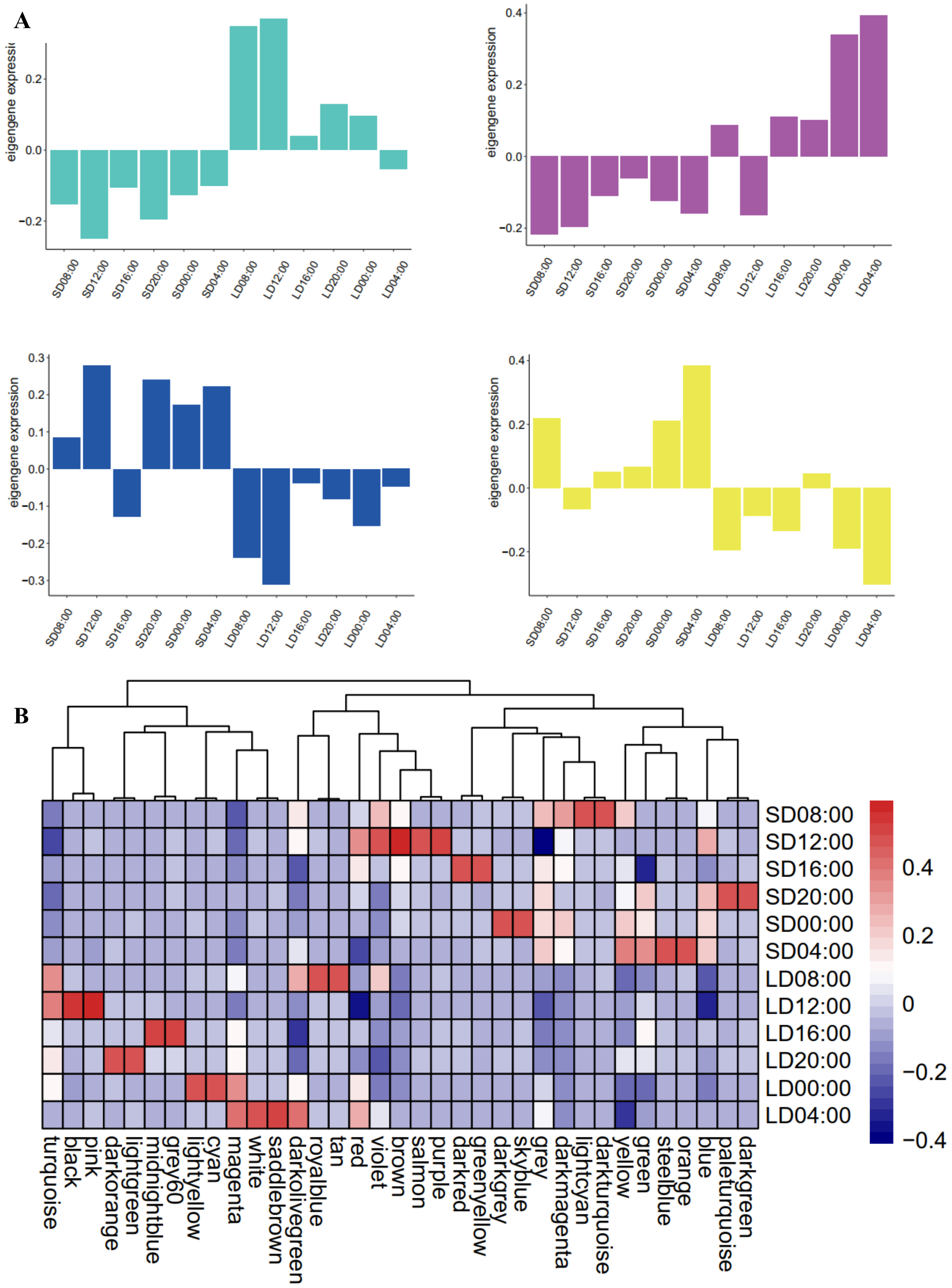

Supplementary Figure 9. (A) Expression pattern of four selected modules (turquoise, magenta, blue and yellow), with high correlation to photoperiod. **(B)**heatmap of all module; Each row corresponds to a time point, Each column corresponds to a module, the color of every cell indicates the eigengene expression.

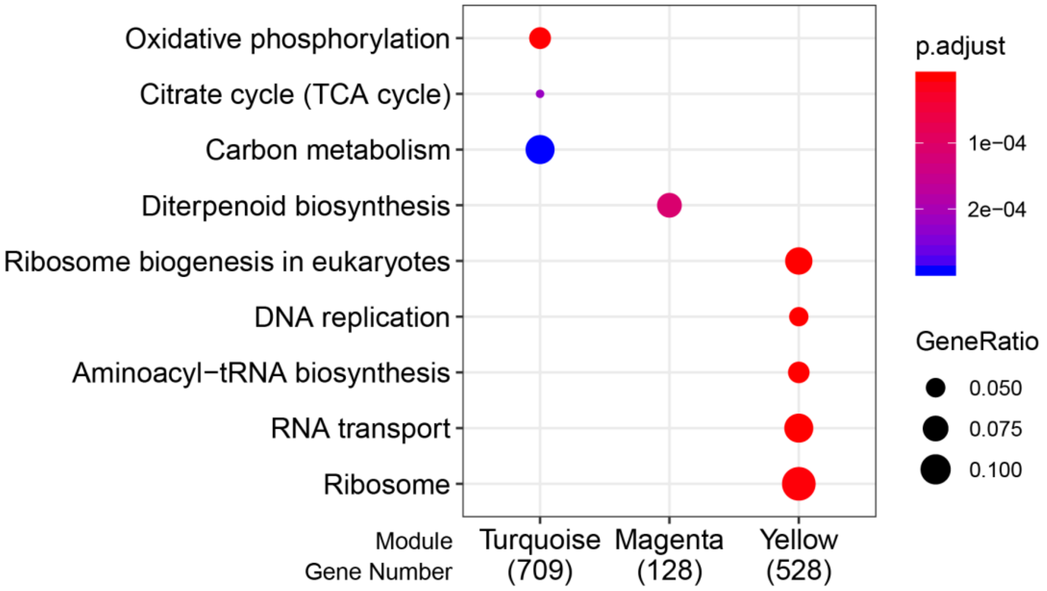

Supplementary Figure 10. KEGG enrichment results of four modules.
X-axis is all time points, Y-axis is various KEGG terms, GeneRatio, depicted by circle size, indicates the rate of gene number of related term compared with all gene number in each time point; p.adjust, shown by color, indicates significance level of enrichment results from blue to red.

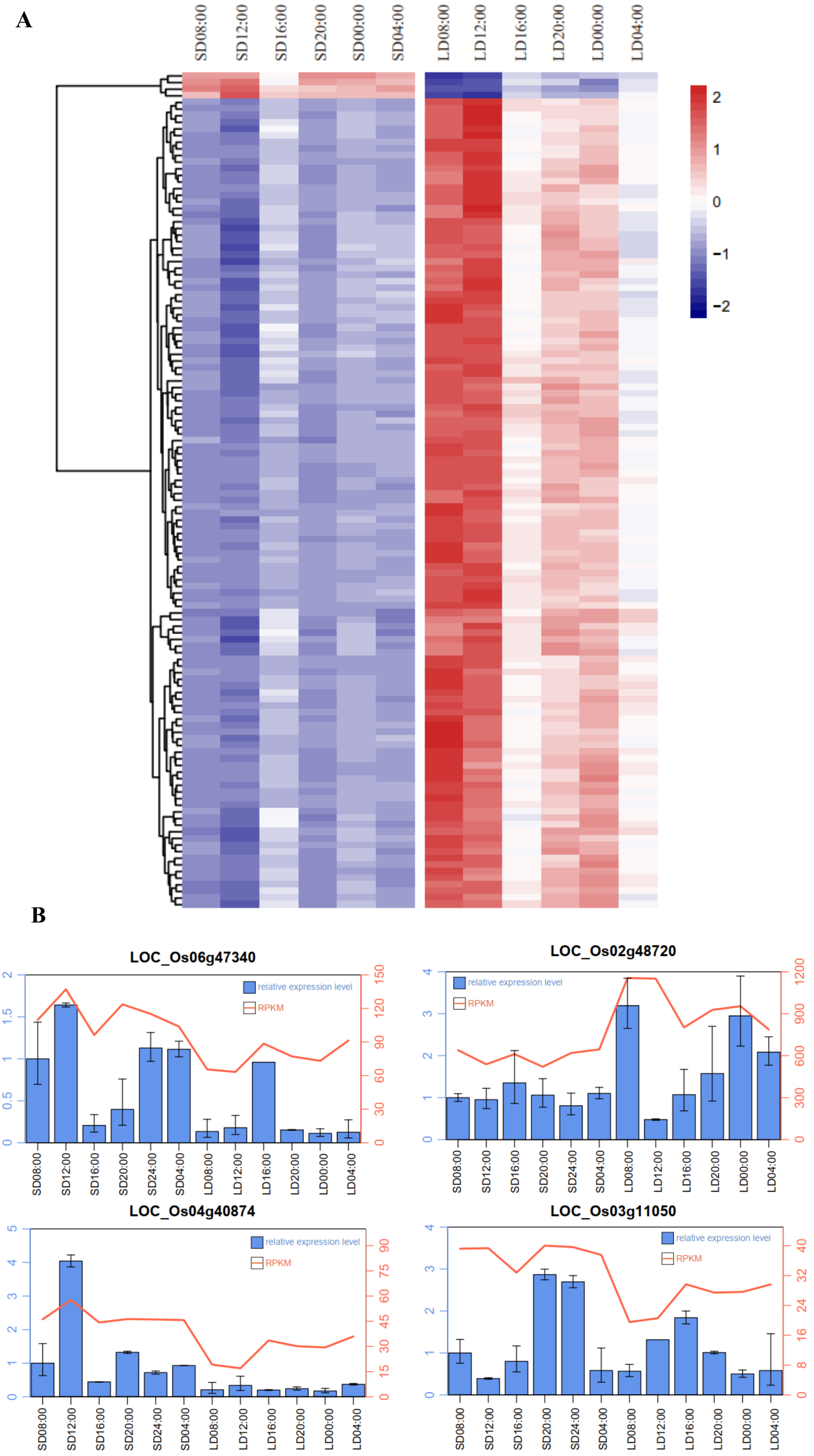

A
SD08:00
SD12:00
SD16:00
SD20:00
SD00:00
SD04:00
LD08:00
LD12:00
LD16:00
LD20:00
LD00:00
LD04:00
2
1
0
−1
−2
B
LOC_Os06g47340
relative expression level
RPKM
LOC_Os02g48720
relative expression level
RPKM
LOC_Os04g40874
relative expression level
RPKM
LOC_Os03g11050
relative expression level
RPKM
SD08:00
SD12:00
SD16:00
SD20:00
SD24:00
SD04:00
LD08:00
LD12:00
LD16:00
LD20:00
LD00:00
LD04:00

Supplementary Figure 11. (A) The heatmap of *CSA*, *Ugp1* and their high correlation genes. cluster tree in the left of heatmap; each row corresponds to a gene, each column corresponds to a time point, the color of every cell indicates the expression level based on z-score normalization. (B) Validation of the expression of co-expressed genes. Gene expression levels were detected by qRT-PCR based on $2^{-\Delta\Delta C}$t method, represents the relative expression level and represents RPKM. The left Y-axis and the blue box are relative expression level, the right Y-axis and the orange line is RPKM.
