# Supplementary material for: Transcriptome Analysis Reveals Photoperiod-Associated Genes Expressed in Rice Anthers
Source: Front Plant Sci. 2021 Feb 26;12:621561. doi: 10.3389/fpls.2021.621561 (PMC7953911; doi:10.3389/fpls.2021.621561)
Supplement: Supplementary file 2 [file Data_Sheet_1.docx]

**Supplementary Table 1** Sequencing statistics of 12 samples with 2 repeats.

High-throughput sequencing obtained 84.7Gb raw data and 681,232,353 reads totally. 81.2Gb (95.6% of raw data) clean data and 608,741,417 clean reads (89.3% of raw reads) were identified after quality filter, each library contains from 22,280,212 to 32,350,561 reads and the average clean reads were 25,364,225. Further, 90.6%-93.1% (average 92.3%) clean reads could be mapped exactly onto rice reference genome while the unique-mapping rate reached at 85.7%-92.7% (average 88.6%).

| SampleName | Raw reads | Raw bases(bp) | clean reads | GC% | MappedRate | UniqueMapped(bp) | UniqueMappedRate |
| --- | --- | --- | --- | --- | --- | --- | --- |
| 04:00(SD)-1 | 33558580 | 4642308114 | 30431816 | 48 | 0.93 | 27623030 | 0.908 |
| 04:00(SD)-2 | 25116365 | 3568619657 | 22351589 | 49 | 0.911 | 19848582 | 0.888 |
| 08:00(SD)-1 | 33524282 | 4271238139 | 29147540 | 50 | 0.934 | 26423049 | 0.907 |
| 08:00(SD)-2 | 26196108 | 3209895757 | 22336664 | 49 | 0.909 | 19592214 | 0.877 |
| 12:00(SD)-1 | 26702929 | 3425181681 | 23488891 | 51 | 0.929 | 20959457 | 0.892 |
| 12:00(SD)-2 | 24859114 | 3241775808 | 22344403 | 51 | 0.918 | 19849940 | 0.888 |
| 16:00(SD)-1 | 25785652 | 3481890688 | 22704181 | 52 | 0.925 | 20342442 | 0.896 |
| 16:00(SD)-2 | 30043559 | 4019331476 | 27119041 | 52 | 0.929 | 24084133 | 0.888 |
| 20:00(SD)-1 | 26685005 | 3341467170 | 23468056 | 50 | 0.924 | 21034933 | 0.896 |
| 20:00(SD)-2 | 35584497 | 4759255337 | 32350561 | 50 | 0.931 | 29171597 | 0.902 |
| 00:00(SD)-1 | 26263782 | 3691941363 | 24124078 | 49 | 0.952 | 22351249 | 0.927 |
| 00:00(SD)-2 | 27440369 | 3476690620 | 24809980 | 51 | 0.932 | 22327575 | 0.9 |
| 04:00(LD)-1 | 27010289 | 3105543921 | 24635451 | 50 | 0.933 | 21704892 | 0.881 |
| 04:00(LD)-2 | 26936245 | 3259879362 | 24088804 | 50 | 0.92 | 20908309 | 0.868 |
| 08:00(LD)-1 | 25510999 | 3169750991 | 23354376 | 50 | 0.933 | 20832912 | 0.892 |
| 08:00(LD)-2 | 30710756 | 3635331846 | 27750468 | 50 | 0.937 | 24891190 | 0.897 |
| 12:00(LD)-1 | 32294671 | 3542411164 | 28147588 | 51 | 0.891 | 24188774 | 0.859 |
| 12:00(LD)-2 | 29579372 | 3322260241 | 26415628 | 50 | 0.899 | 22627173 | 0.857 |
| 16:00(LD)-1 | 28529202 | 3374418664 | 25746089 | 51 | 0.905 | 22465780 | 0.873 |
| 16:00(LD)-2 | 31034744 | 3338252735 | 27684505 | 50 | 0.925 | 23904063 | 0.863 |
| 20:00(LD)-1 | 25092415 | 3193280458 | 22280212 | 51 | 0.906 | 19242474 | 0.864 |
| 20:00(LD)-2 | 27057288 | 3386404619 | 23856147 | 51 | 0.924 | 21066198 | 0.883 |
| 00:00(LD)-1 | 27304551 | 3120251796 | 24629147 | 52 | 0.925 | 21801083 | 0.885 |
| 00:00(LD)-2 | 28411579 | 3211549822 | 25476202 | 52 | 0.917 | 22357162 | 0.878 |

**Supplementary Table 2** The number of genes in the 34 modules

| **Module Colors** | **Freq** | **moduleColors** | **Freq** |
| --- | --- | --- | --- |
| Turquoise | 5852 | Lightyellow | 622 |
| Blue | 4929 | Royalblue | 587 |
| Brown | 3607 | Darkred | 555 |
| Yellow | 2698 | Darkgreen | 554 |
| Green | 2595 | darkturquoise | 515 |
| Red | 2110 | Darkgrey | 492 |
| Black | 1496 | Orange | 487 |
| Pink | 1152 | Darkorange | 474 |
| Magenta | 1132 | White | 472 |
| Purple | 1090 | Skyblue | 453 |
| Greenyellow | 917 | Saddlebrown | 446 |
| Tan | 852 | Steelblue | 445 |
| Salmon | 847 | paleturquoise | 408 |
| Cyan | 710 | Violet | 330 |
| Midnightblue | 703 | Darkolivegreen | 273 |
| Lightcyan | 671 | Darkmagenta | 177 |
| Grey60 | 660 | grey | 78 |
| Lightgreen | 655 |  |  |
